# Heterochronic evolution explains novel body shape in a Triassic coelacanth from Switzerland

**DOI:** 10.1038/s41598-017-13796-0

**Published:** 2017-10-20

**Authors:** Lionel Cavin, Bastien Mennecart, Christian Obrist, Loïc Costeur, Heinz Furrer

**Affiliations:** 1 Department of Geology and Palaeontology, Muséum d’Histoire Naturelle, CP6434, 1211, Geneva, 6 Switzerland; 2 Naturhistorisches Museum Basel, Augustinergasse 2, 4001 Basel, Switzerland; 3 Erliackerweg 8, 4462 Rickenbach, BL Switzerland; 4 Paläontologisches Institut und Museum der Universität Zürich, Karl Schmid-Strasse 4, 8006 Zurich, Switzerland

## Abstract

A bizarre latimeriid coelacanth fish from the Middle Triassic of Switzerland shows skeletal features deviating from the uniform anatomy of coelacanths. The new form is closely related to a modern-looking coelacanth found in the same locality and differences between both are attributed to heterochronic evolution. Most of the modified osteological structures in the new coelacanth have their developmental origin in the skull/trunk interface region in the embryo. Change in the expression of developmental patterning genes, specifically the *Pax1/9* genes, may explain a rapid evolution at the origin of the new coelacanth. This species broadens the morphological disparity range within the lineage of these ‘living fossils’ and exemplifies a case of rapid heterochronic evolution likely trigged by minor changes in gene expression.

## Introduction

Coelacanth fishes, or actinistians, are represented by the living genus *Latimeria* and by about 50 extinct genera ranging from the Early Devonian to the Late Cretaceous. The extant coelacanths are commonly qualified as ‘living fossils’ because of the monotonous morphological disparity they display during their evolutionary history. Indeed anatomically modern coelacanths are known since the Early Devonian^1^ and only a few morphological deviating genera are recorded in the Middle – Late Devonian and in the Early Carboniferous^2^. Here, we describe a coelacanth from the Middle Triassic of Switzerland which shows highly derived anatomical features in the posterior moiety of the skull, the pectoral girdle and the lower jaw. A phylogenetic analysis places the new form as the sister-genus of *Ticinepomis*, a latimeriid found in the same formation. Differences between both genera are attributed to heterochronic evolution. Most of the modified anatomical structures in the new coelacanth have their developmental origin in the skull/trunk interface region in the embryo. Several patterning genes affect this region of the embryo during development^3,4^. Among them, *Pax1/9* genes code for transcription factors required for the development of teeth and skeletal elements of the skull, vertebrae, pectoral girdle and limbs, and a change in their expression may explain a rapid evolution at the origin of the new coelacanth.

Sarcopterygii Romer, 1955

Actinistia Cope, 1891

Latimeriidae Berg, 1940 sensu Dutel *et al*., 2012^5^

*Foreyia* gen. nov.

## Diagnosis

Latimeriid coelacanth with dermal bones covered with numerous large tubercles; hypertrophied otico-occipital portion of skull; fusion of postparietal, supratemporal and extrascapular in postparietal shield, which forms a dome in occipital region; supraorbital sensory canal running in a wide groove; short and curved mandible; pterygopalatine deeper than long with enlarged autopalatine; lachrymojugal and squamosal fused; hypertrophied clavicle; few abdominal vertebrae (seventeen); expanded dorsal and caudal fins; and atrophied pectoral fins.

*Foreyia maxkuhni* gen. et sp. nov.

## Etymology

The generic name honors late Peter L. Forey for his contribution on the study of coelacanth fishes. The specific epithet refers to Max Kuhn, who kindly supported for 12 years the preparation and study of fossils from the Middle Triassic of Graubünden and especially the specimens described here.

## Holotype

**Figure 1:**
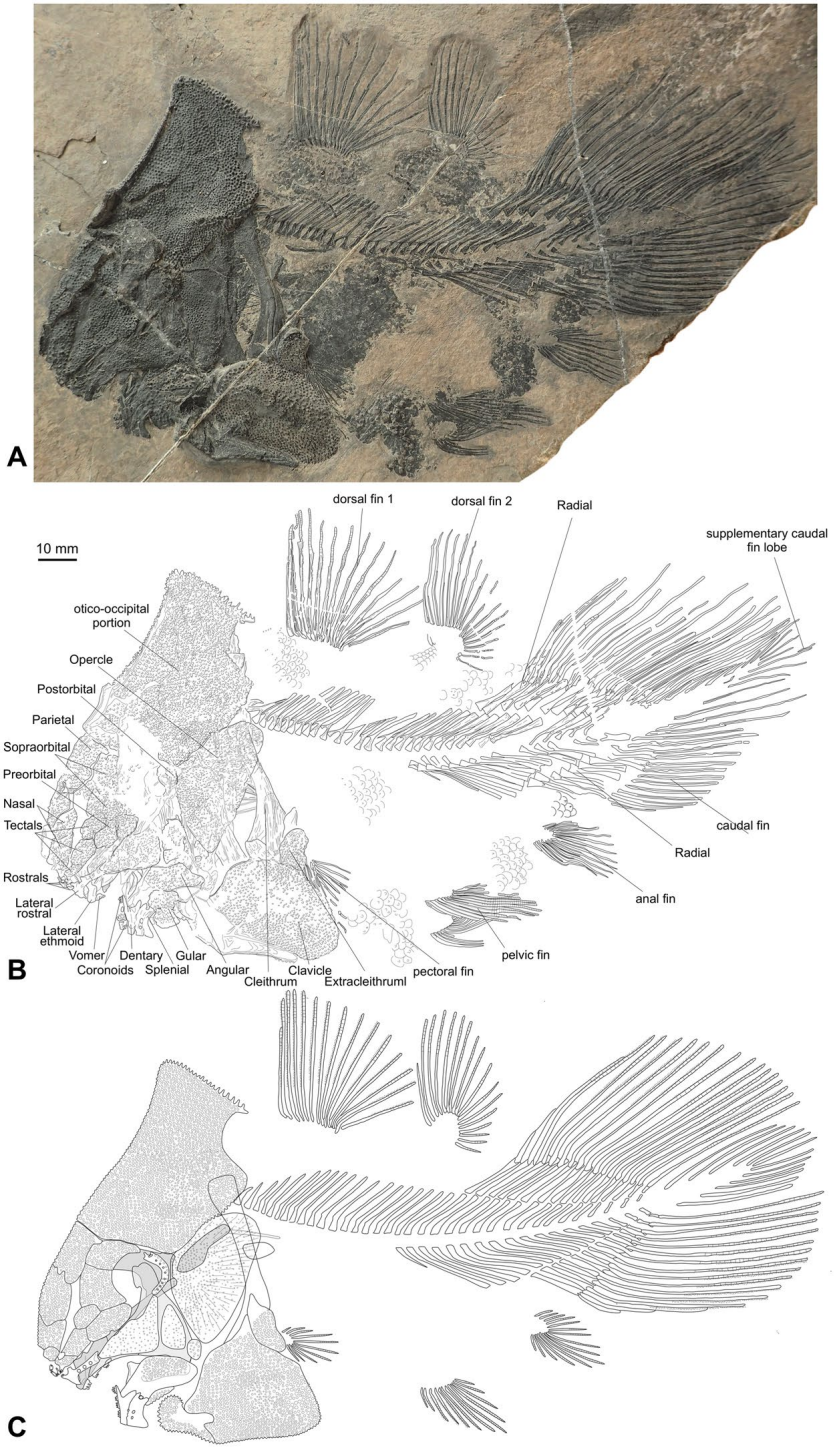
Skeleton of the new coelacanth *Foreyia maxkuhni* gen. et sp. nov. (**A**) Photo and (**B**) outline of the holotype (PIMUZ A/I 4620). (**C**) Reconstruction of the whole skeleton.

**Figure 2:**
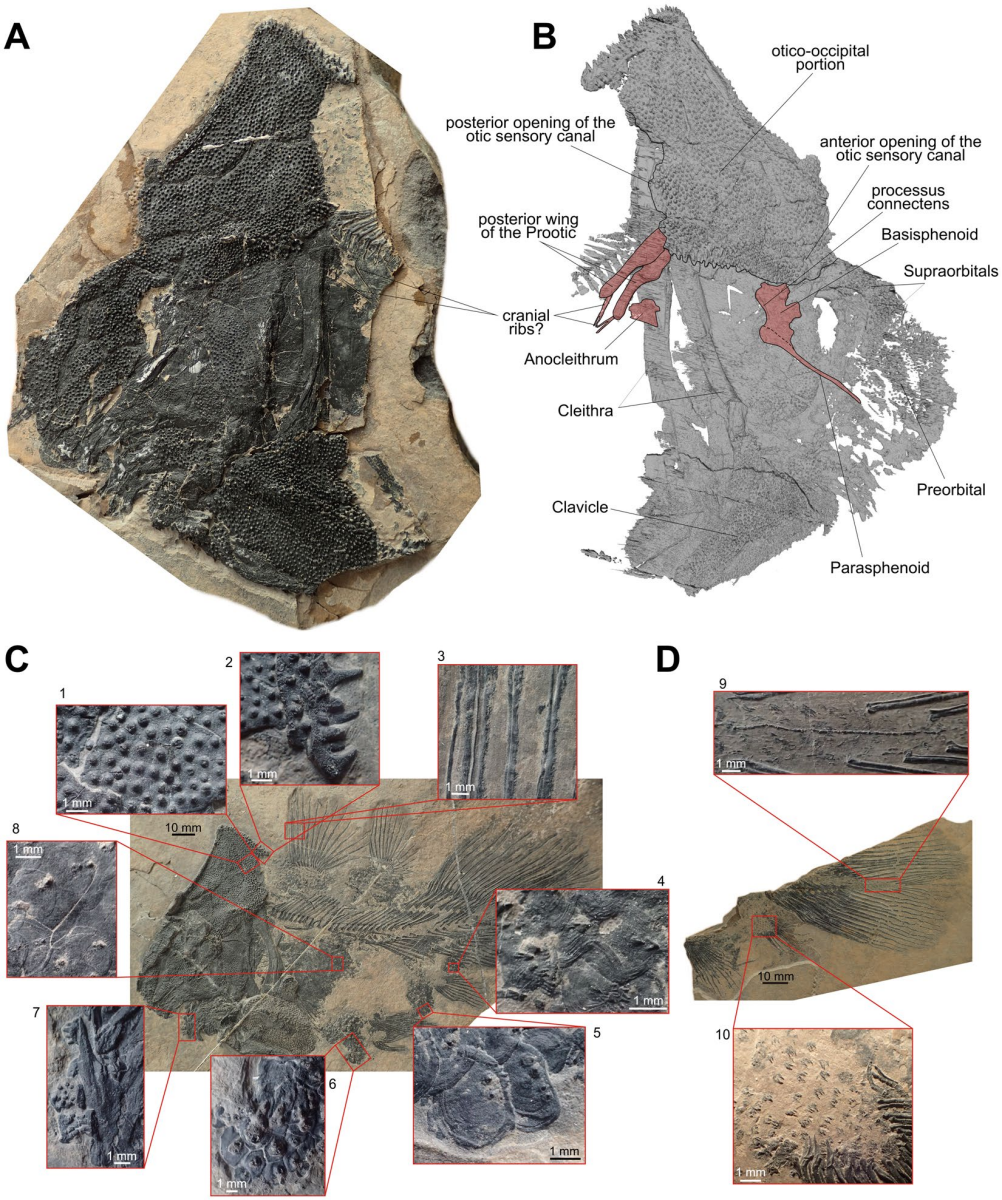
Osteological details of the new coelacanth *Foreyia maxkuhni* gen. et sp. nov. (**A**) Photo and (**B**) surface CT reconstruction of the skull of the paratype (PIMUZ A/I 4372). (**C**) Tubercles and denticles in the Holotype (PIMUZ A/I 4620) and (**D**) in the paratype (PIMUZ A/I 4372). 1, tubercles on the skull roof. 2, large spine-like tubercles on the posterior margin of the otico-occipital shield. 3, denticles on the fin rays of the first dorsal fin. 4, scales with denticles from the ventral margin of the caudal peduncle. 5, scales with denticles from the anal region. 6, scales with denticles from the belly region. 7, toothed coronoid bones. 8, scales with denticles from the flank. 9, supplementary caudal fin lobe with spiny scales. 10, Scales with denticles from the lobe of the anal fin.

A complete specimen preserved in left lateral view (PIMUZ A/I 4620) (Figs 1, 2C, S2, S4, S6).

## Paratype

A specimen comprising the head and the tail in left lateral view (PIMUZ A/I 4372) (Figs 2A,B,D, S3, S5, S6).

## Type locality and horizon

Site DF 4 near the Ducanfurgga (Graubünden, Switzerland), upper part of the Prosanto Formation, Middle Triassic (early Ladinian, 240.91 million years ago)^6,7^ (Fig. S1).

## Diagnosis

As for genus, single species.

## Description

A detailed description of *Foreyia* is available in online content (Supplementary Information and Figs S2–S6). Only features departing from generalized coelacanths are mentioned herein. The skull roof of the posterior part of the otico-occipital portion of the neurocranium is circa 1.5 times longer than the skull roof of the ethmosphenoid portion. In most actinistians, the ethmosphenoid portion is significantly longer, between 1.5 to 2 times, than the otico-occipital portion. In a few Palaeozoic genera, the otico-occipital portion is almost as long as the ethmosphenoid portion (*Caridosuctor*, *Rhabdoderma*) or is even slightly longer (*Miguashaia*, *Diplocercides*, *Sassenia*), but never in the proportions seen in *Foreyia* (1.5 times longer). All the bones of the skull roof, the angular bone in the lower jaw and the clavicles are covered with densely packed large tubercles, while cheek and opercular bones are covered with smaller and less densely packed tubercles (Fig. 2C). *Foreyia* is unique among coelacanths by its proportionally huge postparietal shield, which forms a dome in this fish and mirrors the ventral hypertrophied clavicle. No limits between ossifications are visible within the postparietal shield, neither with optical instruments nor with CT images (Figs 2B, S5, Smovie). We hypothesize that the postparietal shield is composed of a single, paired or unpaired ossification resulting from the complete fusion of the original ossifications (postparietals, supratemporals and extrascapulars). The skull roof of the parietonasal shield of *Foreyia* is typical for coelacanths, except the supraorbital sensory canal, which ran in a wide groove between the medial and the lateral series of bones and the ethmoid region, which is short. CT images of the paratype (PIMUZ A/I 4372) shows embedded in the matrix two rounded processes extending posteriorly from the postparietal shield and overpassing posteriorly the cleithra (Figs 2B, S5). They are interpreted as the posterior wings of the prootics, which have shifted backward before fossilization. These wings are associated with rod-like elements visible externally on the paratype that we tentatively identify as cranial ribs. A large triangular plate-like bone in the cheek is interpreted as a fused lachrymojugal and jugal. The lower jaw of *Foreyia* has an unusual general comma-shape, but the typical actinistian apomorphic organization is recognized (Fig. S6). The dentary is hooked-shaped as in *Latimeria* and other derived coelacanths.

The shoulder girdle of coelacanths is said to be remarkably conservative, except in *Miguashaia* ^8^ and, now, *Foreyia*. Contrary to all other coelacanths, which show a gap between the skull and the pectoral girdle, the cleithrum in *Foreyia* is situated at the level of the otico-occipital moiety. CT images show the dorsal extremities of the cleithra positioned against the postparietal shield (Figs 2B, S5), but the exact nature of the connection between the pectoral girdle and the skull cannot be observed. No anocleithra are visible externally, but the CT scan shows in the matrix a paired ossification oriented posteriorly and located on the internal side of the cleithrum in the mid-depth of the vertical branch (Fig. 2B; Fig. S5). Although the shape and the location are unusual for coelacanths, these bones are regarded as modified anocleithra. The ventral half of the cleithrum is hidden under the hypertrophied clavicle completely covered with the same strong ornamentation as present on the skull roof. A reniform extracleithrum covered by the same kind of tubercles borders a concavity of the posterodorsal corner of the clavicle. Its large ovoid shape is more reminiscent of the extracleithrum of the basal *Miguashaia* rather than that of the more derived genera, in which it is much slender^9,10^. A probable interclavicle is fused through a V-shaped suture to the anteroventral tips of both clavicles. Most coelacanths have no interclavicle, except *Whitheia* and *Laugia*, in which it is a small subdermal ossification of probable endochondral origin^8^, and *Miguashaia*, in which it bears ornamentation and has a dermal origin^10^. The scales bear two to four spines and those from the belly seem to form a paving-like structure, which may have acted as a kind of armoured protection. The postcranial skeleton of *Foreyia* fits the general *Bauplan* of coelacanths, except meristic features and fin size proportions. The paired fins are characterized by low number of fin rays: ten rays in the pectoral fins (only *Allenypterus* has less rays (9)) and 12 rays in the pelvic fins (*Allenypterus* has less rays (6) and *Hadronector* has the same number). To the contrary, the dorsal and caudal fins are proportionally overdeveloped in *Foreyia*. The numbers of rays in these fins are in the range of other coelacanths, except for the anterior dorsal, which has the highest number together with *Allenypterus* (15). The total number of vertebrae is the lowest known among coelacanths due to an unusually low number of abdominal vertebrae (17).

## Discussion

### Phylogenetic relationships

**Figure 3:**
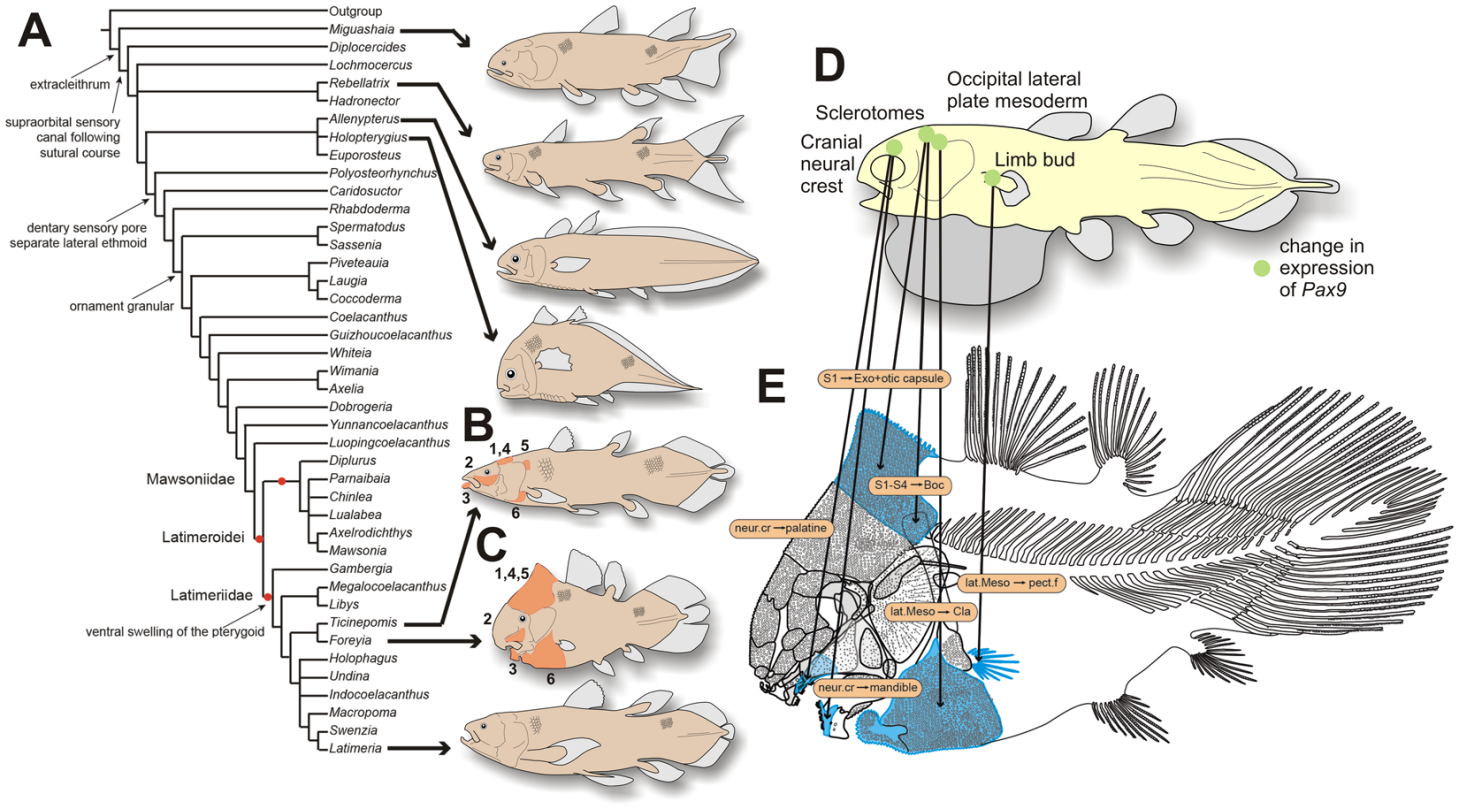
Phylogenetic relationships of *Foreyia maxkuhni* gen. et sp. nov. and developmental origin of the derived characters. (**A**) Strict consensus trees of the 259 most parsimonious trees of 317 steps (CI = 0.3817, RI = 0.6766) with some of the uniquely derived characters present in *Foreyia maxkuhni* on the left, and reconstructions of genera with atypical general morphology. (**B** and **C**) Shared features of *Ticinepomis peyeri* and *Foreyia maxkuhni* (in orange) not included in the cladistics analysis (see main text for numbers). (**D**) Reconstruction of a coelacanth embryo with localization of embryonic tissues that give rise the derived skeletal features present in *Foreyia*. It is hypothesized that changes in the expression of *Pax9* may have altered the derived characters shown in blue on the reconstruction (**E**). All the drawings were made by LC. Abbreviation: Boc, basioccipital; Cla, clavicle; Exo, exoccipital; lat. Meso., lateral mesoderm; neur. cr., neural crest; pect. f., pectoral fin; S (numbered), somite.

At first sight, the highly-modified coelacanth *Foreyia* recalls basal Palaeozoic coelacanths. In particular, its general head morphology and some meristic features are reminiscent of the Carboniferous *Allenypterus*, such as a steep and convex profile of the anterior moiety in lateral view and a proportionally short and deep mandible. Its pectoral girdle shares superficial characters with the Devonian *Miguashaia*. However, a cladistic analysis places *Foreyia* as the sister-taxon of *Ticinepomis* ^11^, a genus recovered from the same formation at a nearby locality^12^ (Figs 3A, S7). Both genera are nested within the latimeriids. The node supporting the *Latimeria* – *Foreyia* clade is weakly supported but *Ticinepomis* shares with *Foreyia* other characters not included in the cladistic analysis (Fig. 2B,C). These are: 1) The postparietal shield of *Ticinepomis* is proportionally smaller than in *Foreyia*, but no sutures are visible between the postparietal and supratemporal ossifications as in *Foreyia*; 2) The lachrymojugal and squamosal are poorly preserved and fragmented in the holotype of *T. peyeri*. A possible reconstruction based on direct observation of the holotype is to regard these fragments as belonging to a single large triangular plate corresponding to the fusion of the lachrymojugal and squamosal, as in *Foreyia*; 3) The lower jaw of *Ticinepomis* is less derived than that of *Foreyia*. However, the dentary and the splenial of the former are both angled, reminiscent of the curved mandible of the latter; 4) The ornamentation of most of the dermal bones consists in both genera of tubercles, although in *Ticinepomis* they are smaller; 5) A broad dorsal extremity of the cleithrum is present in both genera; 6) A massive ornamented clavicle is present in both genera, but in a much more important proportion in *Foreyia* than in *Ticinepomis*.

### Heterochronic evolution and its developmental basis

**Figure 4:**
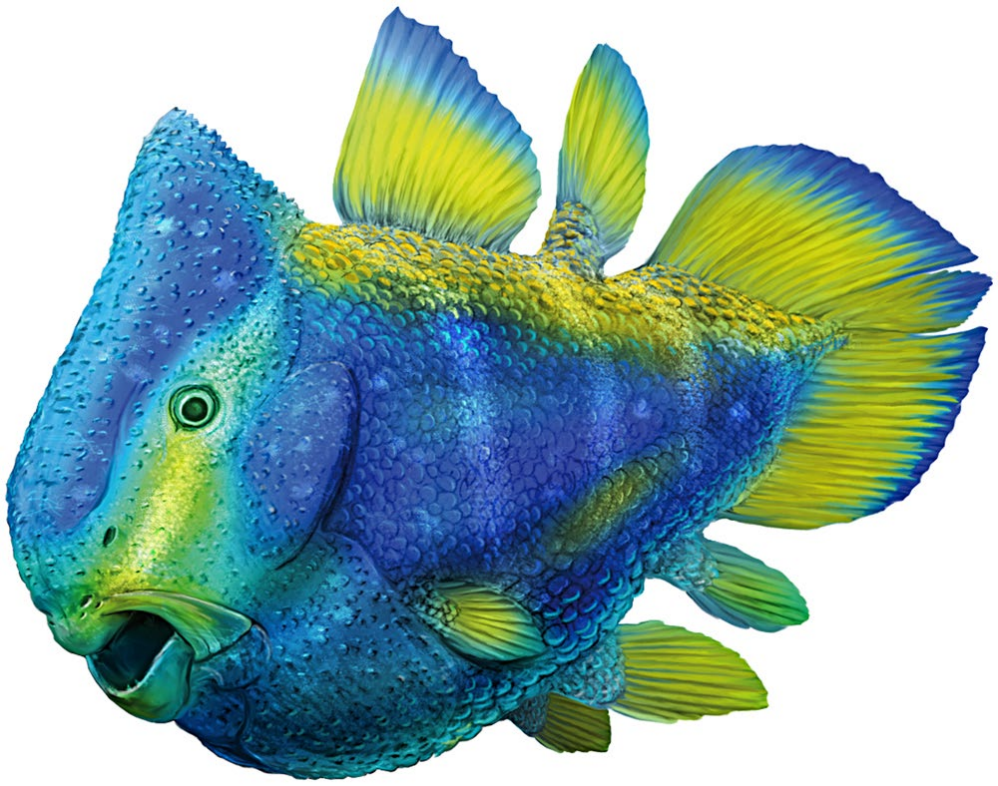
Reconstruction of the living coelacanth *Foreyia maxkuhni* gen. et sp. nov. Artwork by Alain Bénéteau.

Most of the shared features in *Ticinepomis* and *Foreyia* are more weakly developed in the former than in the latter genus, and they indicate a possible heterochronic evolution at the origin of *Foreyia*. This hypothesis is strengthened by the fact that the general coelacanth skeletal organization is not altered in *Foreyia*, but only relative bone sizes vary compared to the generalized coelacanths *Bauplan* (hypertrophied occipital and clavicular regions, comma-shaped mandible, few abdominal vertebrae and rays in paired fins, and dense covering of large tubercles on the dermal bones and denticles on the scales). Several of these features are developmentally linked in sarcopterygians and, compared with extant models, partly under the control of the same genes. In the chick embryo, the anterior most somites give rise to part of the otic capsule and the exoccipital bone (somite 1) and to the basioccipital bone (somites 2–4)^13^. The occipital lateral plate mesoderm at the level of somites 1–3 gives rise to the ventromedial extremity of the clavicle in amniotes, which is regarded in part as homologous to the dermal clavicle of bony fishes^3,4^. Although numerous developmental patterning genes have a control on these features, the best candidate is the paired box gene 9, or *Pax9*, widely distributed among vertebrates and present in *Latimeria* ^14,15^ (alternative genes, such as *Prrx1*/*Prrx2*, *HoxD*, *Tbx14* are discussed in Supplementary Information). In extant bracketing clades of coelacanths, chondrichthyans and amniotes, the embryonic expression of *Pax9* occurs at the level of the head mesoderm, of the sclerotomes (those from the first somites give rise to the occipital bones), of the postotic mesoderm (gives rise to the clavicle) and of the trunk mesoderm (gives rise to paired limbs), as well as at the level of the neural crest (give rises to odontodes)^16,17^. *Pax9* expression on the neural crest at the level of the first rhombomeres also affects the palatine and the coronoid regions in the mouse^16^, two anatomical domains also modified in *Foreyia*. Although *Pax9* in deficient mice does no show phenotypic features directly linkable to the peculiar morphology of *Foreyia*, the targeted embryological tissues make this gene potentially at the origin of its heterochronic evolution (Fig. 3D,E). *Pax9* regulates synergetically the development of the vertebral column with *Pax1*. The latter has a more limited expression than *Pax9* in amniotes and has an effect on the development of the pectoral girdle, particularly on the acromion, which is a process on the scapula connecting the clavicle^18,19^. The acromion is mesodermal in origin^3^, as is the hypertrophied clavicle of *Foreyia*. It is possible that in coelacanths the expression *Pax1* and *Pax9* are more similar between them than they are in amniotes, as it is the case in the ray-fin fish Medaka^20^. In this case, both genes should be considered together in their effects on the phenotype. The search of a single genetic source is an oversimplification since we know that *Pax* genes work in cooperation with *Hox* genes^21,22^. The developmental and genetic pathways proposed here suggest that the bizarre morphology of *Foreyia* (Fig. 4) might be the consequence of a rapid heterochronic evolution.

## Methods

### Fossil preparation

Both new specimens of coelacanth were found during systematic bed-by-bed excavations in the upper part of the Prosanto Formation by Christian Obrist, under direction of Heinz Furrer. Both specimens were found broken in several fragments, then glued together and very carefully prepared mechanically with air-tool, fine sharp steel needles and sand-blaster by C.O.: The holotype (PIMUZ A/I 4620), a complete skeleton found in summer 2014 (bed 141) and prepared in 2016 during 150 hours; the paratype (PIMUZ A/I 4372), a broken specimen recovered in two fragments in summer 2015 (bed 150) and prepared in 2015 during 30 hours.

### Computed tomography

The paratype (PIMUZ A/I 4372) of *Foreyia maxkuhni* was scanned with high resolution x-ray computed tomography at the Biomaterial Science Center of the University of Basel using a phoenix nanotom® (General Electric Wunstorf, Germany) equipped with a 180 kV/15 W nanofocus x-ray source. A voltage of 180 kV and current of 30 mA were used with a 0.25-mm Cu filter. 1440 poses were taken with an average of 6 images for each pose.

### Phylogenetic analysis

We ran the analysis using PAUP 4.0b10^23^ heuristic search option, random addition sequence, replicated 100 times, 10 trees held at each iteration, and tree bisection and reconnection branch swapping.

### Data Availability

The protocols used in the development of this study are available in the ‘Supplementary Information’ section.

## Electronic supplementary material

Supplementary Information

Supplementary Video
