# Supplementary material for: Heterochronic evolution explains novel body shape in a Triassic coelacanth from Switzerland
Source: Sci Rep. 2017 Oct 20;7:13695. doi: 10.1038/s41598-017-13796-0 (PMC5651877; doi:10.1038/s41598-017-13796-0)
Supplement: Supplementary file 1 — Supplementary Information [file 41598_2017_13796_MOESM1_ESM.pdf]

Supplementary Information for:

Heterochronic evolution explains novel body shape in a Triassic coelacanth from Switzerland

Lionel Cavin[1]*, Bastien Mennecart[2], Christian Obrist[3], Loïc Costeur[2], Heinz Furrer[4]

[1]Department of Geology and Palaeontology, Muséum d'Histoire Naturelle, CP6434, 1211 Geneva 6, Switzerland.

[2]Naturhistorisches Museum Basel, Augustinergasse 2, 4001 Basel, Switzerland.

[3]Erliackerweg 8, 4462 Rickenbach, BL, Switzerland.

[4]Paläontologisches Institut und Museum der Universität Zürich, Karl Schmid-Strasse 4, 8006 Zurich, Switzerland.

*Correspondence to: lionel.cavin@ville-ge.ch

A – Supplementary Figures 1-7

B – Geological settings

C – Supplementary description of the osteology of *Foreyia maxkuhni* gen. et sp. nov.

D – Relationships, character definitions and datamatrix for phylogenetic analysis

E – Heterochronic development in *Foreyia maxkuhni* gen. et sp. nov. and its potential genetic roots

F – Supplementary Movie

A- Supplementary Figures

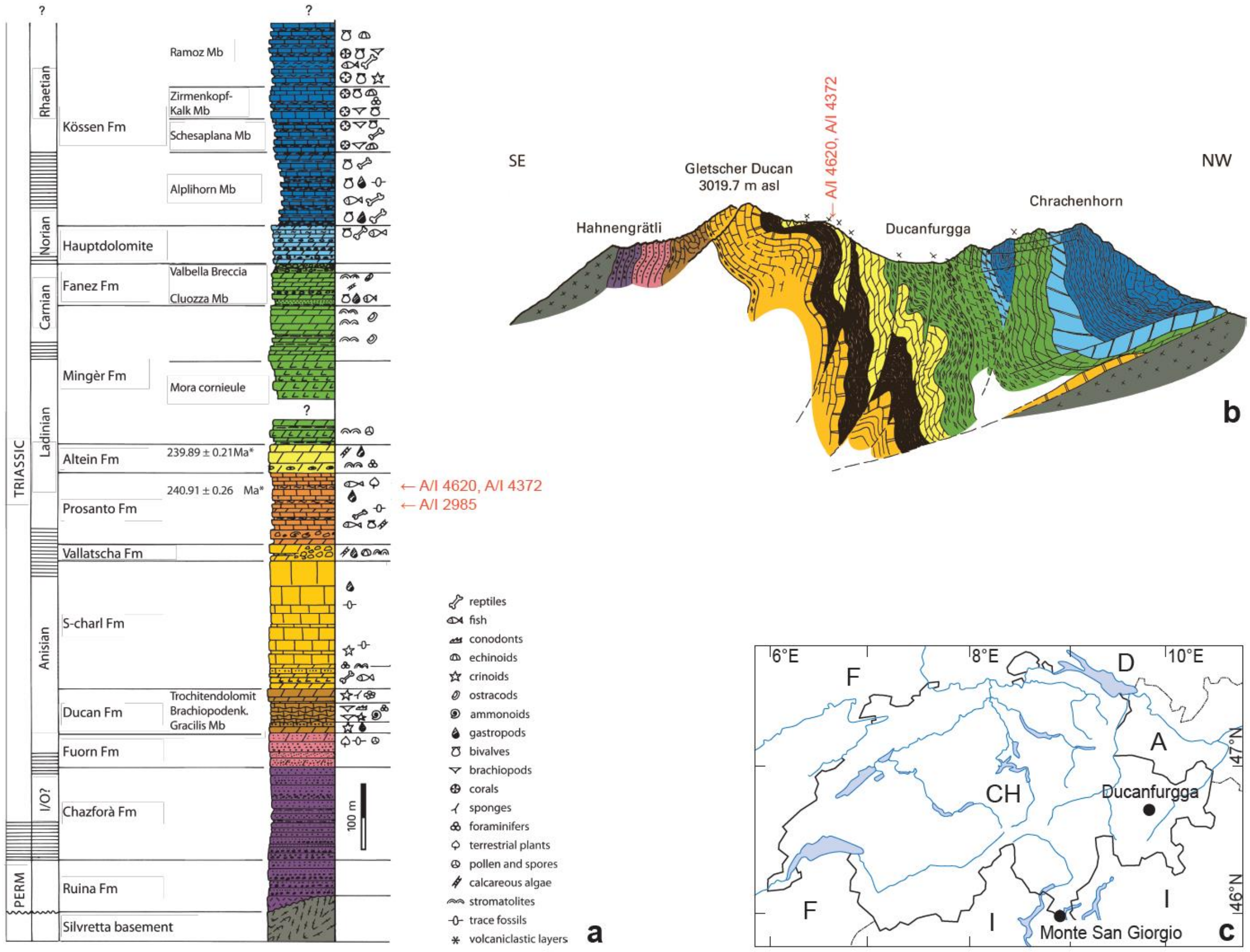

Figure S1. Locality and stratigraphic position of the Ducanfurgga site near Davos with the coelacanths (Graubünden, south-eastern Switzerland). **a**, Stratigraphic column at Ducanfurgga with localization of the U-Pb zircon ages of volcaniclastic layers, of both specimens of *Foreyia maxkuhni*, gen. et sp. nov. (holotype PIMUZ A/I 4620, paratype PIMUZ A/I 4372), and of the specimen of *Ticinepomis* cf. *T. peyeri* (PIMUZ A/I 2985). **b**, Geological section at Ducanfurgga (Upper Austroalpine Silvretta Nappe). **c**, Map showing the Ducanfurgga locality relative to the World

Heritage vertebrate site of Monte San Giorgio (Software: ® Adobe Illustrator CS6, Version 16.0.3, http://www.adobe.com/).

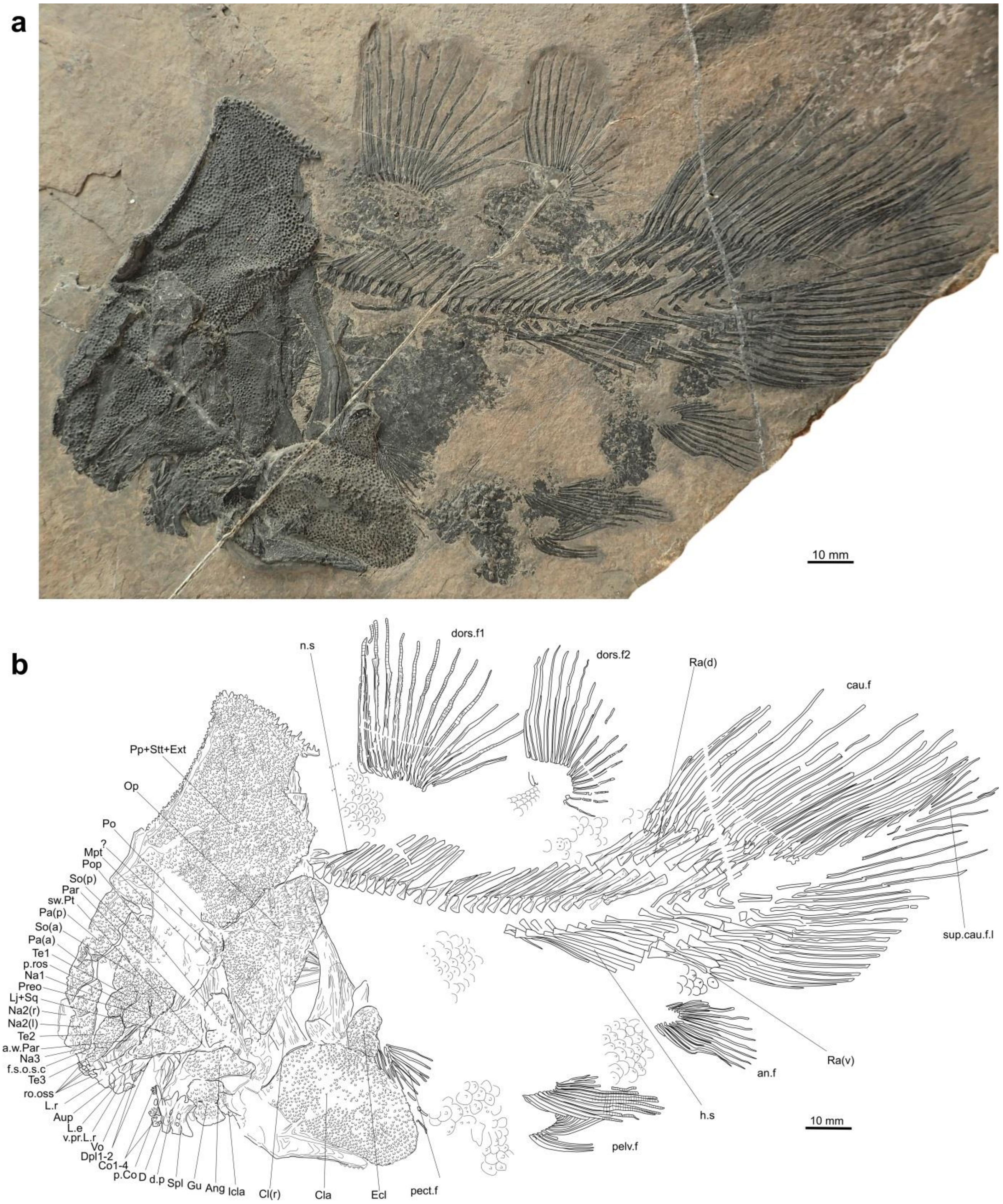

Figure S2. ***Foreyia maxkuhni*, gen. et sp. nov. a**, Photograph and drawing (**b**) of the holotype (PIMUZ A/I 4620). (a), anterior; Ang, angular; Aup, autopalatine; a.w.Par, anterior wing of the parasphenoid; cau.f, caudal fin; Cl, cleithrum; Cla, clavicle; Co, coronoids (numbered); (d), dorsal; De, dentary; dor.f, dorsal fin (numbered); d.p, enlarged sensory pore within the dentary; Dpl, dermopalatine (numbered); Ecl, extracleithrum; f.s.o.s.c, foramen of the supraorbital sensory canal; Gu, gular plate; h.s, haemal spine; Icla, interclavicle; L.e, lateral ethmoid; L.r, lateral rostral; Lj+Sq, lachrymojugal + squamosal; Mpt, metapterygoid; n.s, neural spine; Op, opercle; (p), posterior; Pa, parietal; Par, parasphenoid; p.Co, principal coronoid; pect.f, pectoral fin; pelv.f, pelvic fin; Po, postorbital; Pop, preopercle; Pp+Stt+Ext, ossification corresponding to the area occupied by the postorbital, supratemporal and extrascapulars in other actinistians; Preo, preorbital; Ra, radial; ro.oss, rostral ossicles; So, supraorbital; Spl, splenial; sup.cau.f.l, supplementary caudal fin lobe; sw.Pt, ventral swelling of the pterygoid; Te, tectal; (v), ventral; Vo, vomer; v.pr.L.r, ventral process of the lateral rostral.

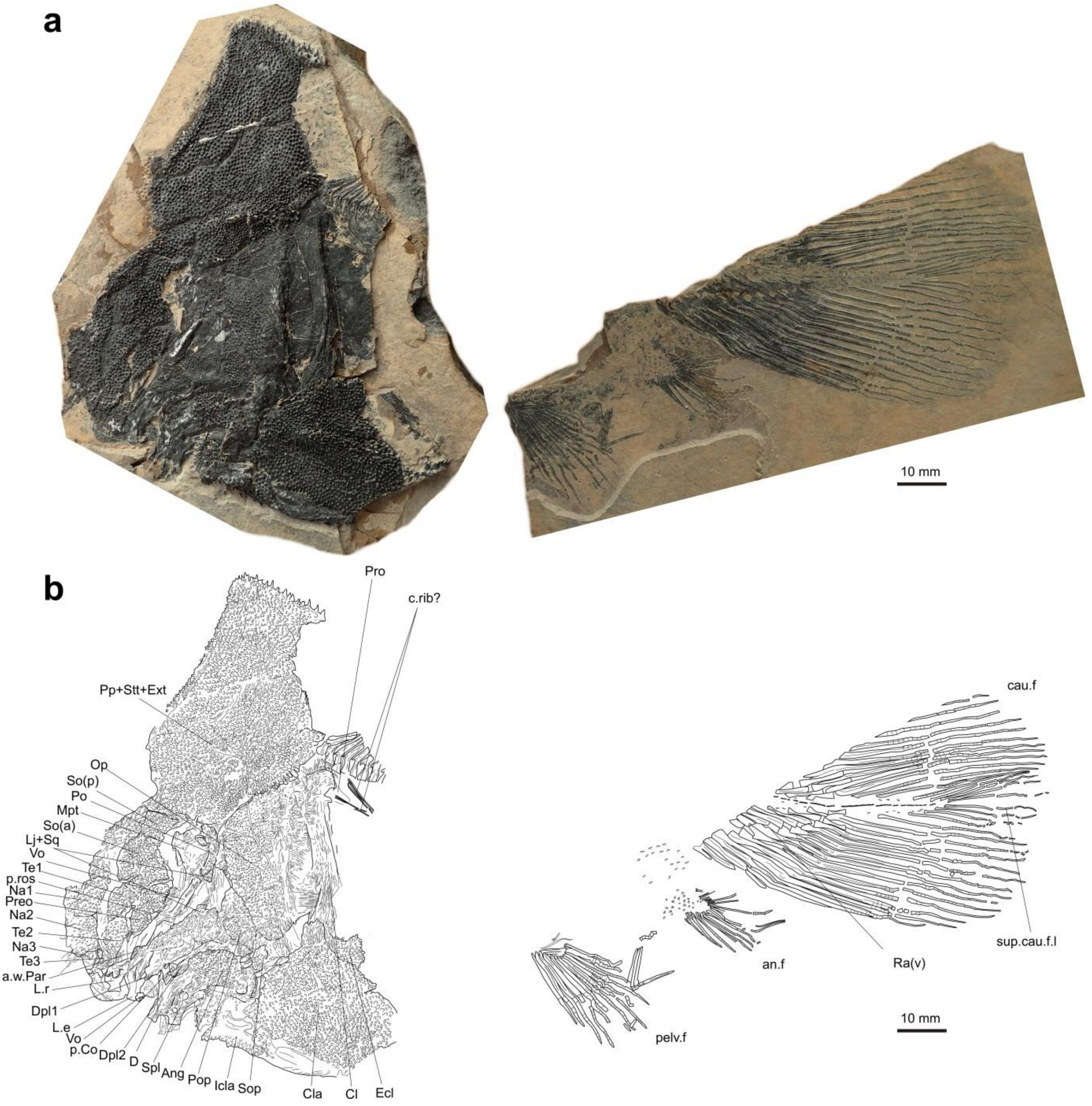

Figure S3. ***Foreyia maxkuhni*, gen. et sp. nov. a**, Photograph and drawing (**b**) of the paratype (PIMUZ A/I 4372). (a), anterior; Ang, angular; a.w.Par, anterior wing of the parasphenoid; cau.f, caudal fin; Cl, cleithrum; Cla, clavicle; Co, coronoids (numbered); c.rib?, possible cranial rib; (d), dorsal; De, dentary; Dpl, dermopalatine (numbered); Ecl, extracleithrum; Gu, gular plate; Icla, interclavicle; L.e, lateral ethmoid; L.r, lateral rostral; Lj+Sq, lachrymojugal + squamosal; Mpt, metapterygoid; Na, nasal; Op, opercle; (p), posterior; p.Co, principal coronoid; pelv.f, pelvic fin; Po, postorbital; Pop, preopercle; Pp+Stt+Ext, ossification corresponding to the area occupied by the postorbital, supratemporal and extrascapulars in other actinistians; pr.con, processus connectens; Preo, preorbital; Pro, prootic; p.ros, posterior opening of the rostral organ; Ra, radial; Rart,

retroarticular; Ro, rostral; So, supraorbital; Sop, subopercle; Spl, splenial; sup.cau.f.l, supplementary caudal fin lobe; Te, tectal; (v), ventral; Vo, vomer.

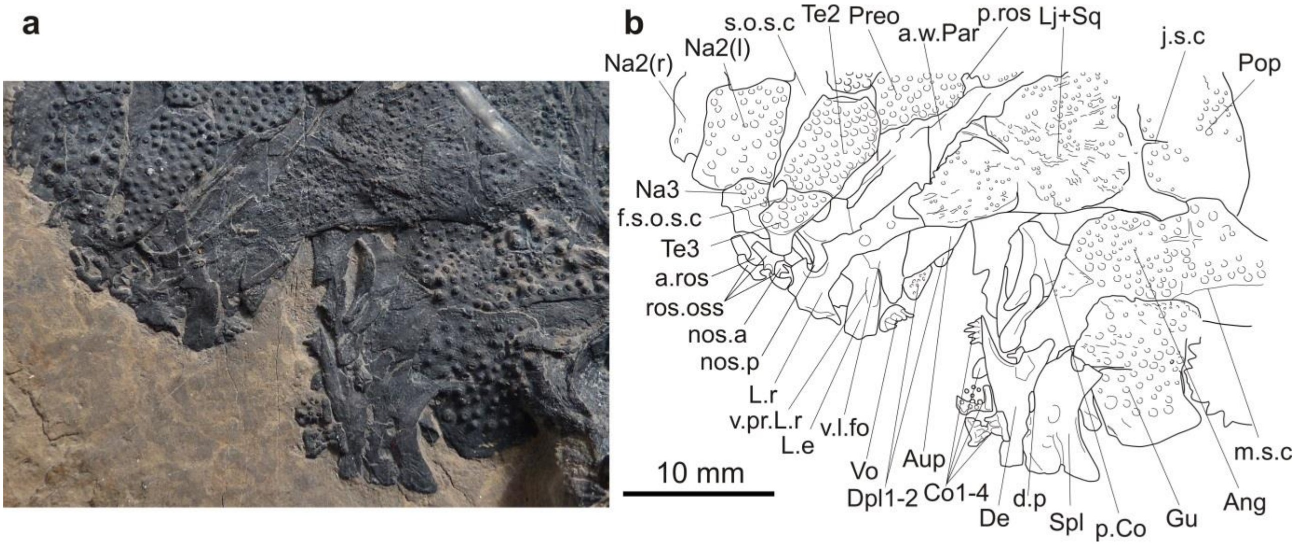

Figure S4. ***Foreyia maxkuhni*, gen. et sp. nov. a**, detail of the anterior part of the skull of the holotype (PIMUZ A/I 4620) with interpretative drawing (**b**). Ang, angular; a.ros, opening of the anterior rostral organ; Aup, autopalatine; a.w.Par, anterior wing of the parasphenoid; Co, coronoids (numbered); De, dentary; d.p, enlarged sensory pore within the dentary; Dpl, dermopalatine (numbered); f.s.o.s.c, foramen of the supraorbital sensory canal; Gu, gular plate; L.e, lateral ethmoid; L.r, lateral rostral; Lj+Sq, lachrymojugal + squamosal; j.s.c, jugular sensory canal; m.s.c, mandibular sensory canal; Na, nasal; nos.a, anterior nostril; nos.p, posterior nostril; p.Co, principal coronoid; Pop, preopercle; Preo, preorbital; p.ros, posterior opening of the rostral organ; ro.oss, rostral ossicles; s.o.s.c, supraorbital sensory canal; Spl, splenial; Te, tectal (numbered); V.l.fo, ventrolateral fossa; Vo, vomer; v.pr.L.r, ventral process of the lateral rostral.

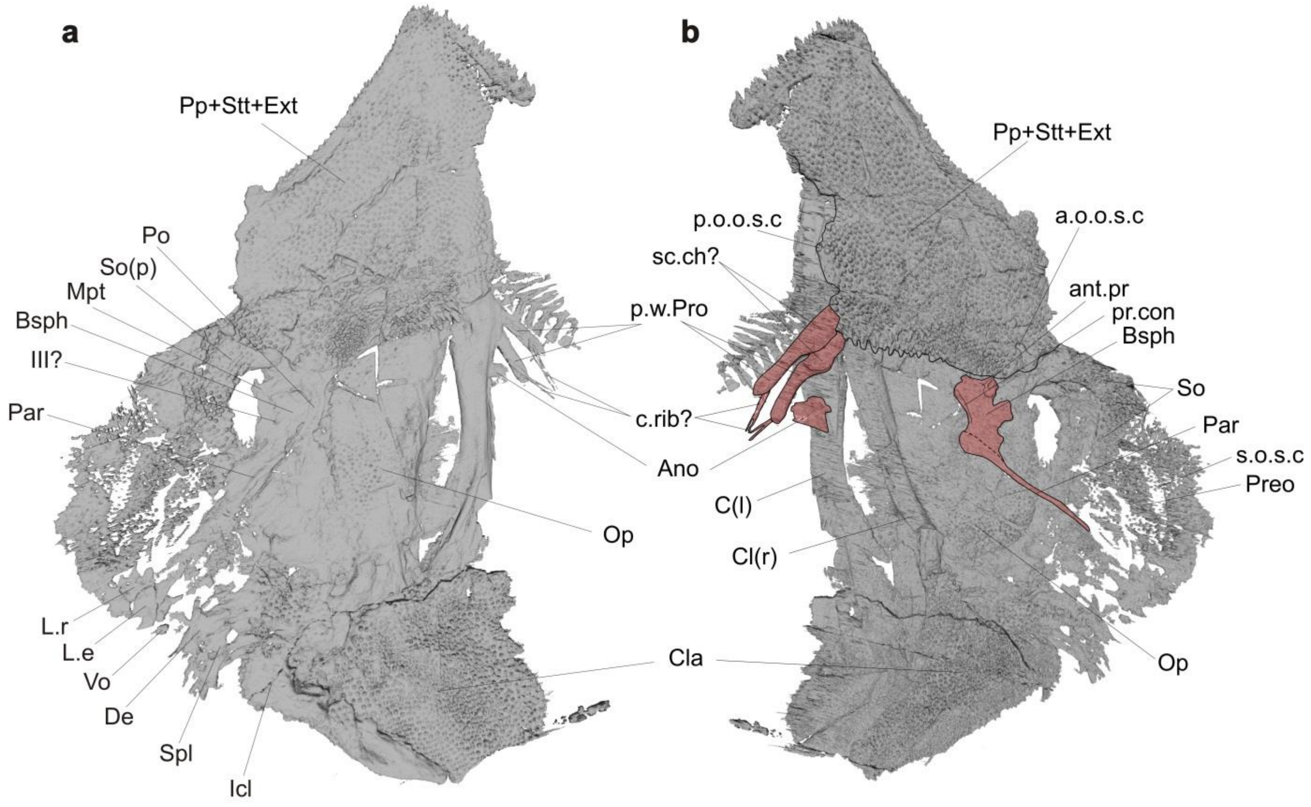

Figure S5. *Foreyia maxkuhni*, gen. et sp. nov., surface CT image of the paratype (PIMUZ A/I 4372). **a**, left side (visible externally). **b**, right side (in the matrix). (See also Supplementary Data). Ano, anocleithrum; a.o.o.s.c, anterior opening of the otic sensory canal; Bsph,basisphenoid; Cl, cleithrum; Cla, clavicle; c.rib?, possible cranial rib; De, dentary; L.e, lateral ethmoid; L.r, lateral rostral; Mpt, metapterygoid; Op, opercle; (p), posterior; Par, parasphenoid; Po, postorbital; p.o.o.s.c, posterior opening of the otic sensory canal; Pp+Stt+Ext, ossification corresponding to the area occupied by the postorbital, supratemporal and extrascapulars in other actinistians; pr.con, processus connectens; Pp.w.Pro, posterior wing of the prootic; Preo, preorbital; sc.ch?, possible saccular chamber; So, supraorbital; s.o.s.c, supraorbital sensory canal; Spl, splenial; III?, possible oculomotor foramen.

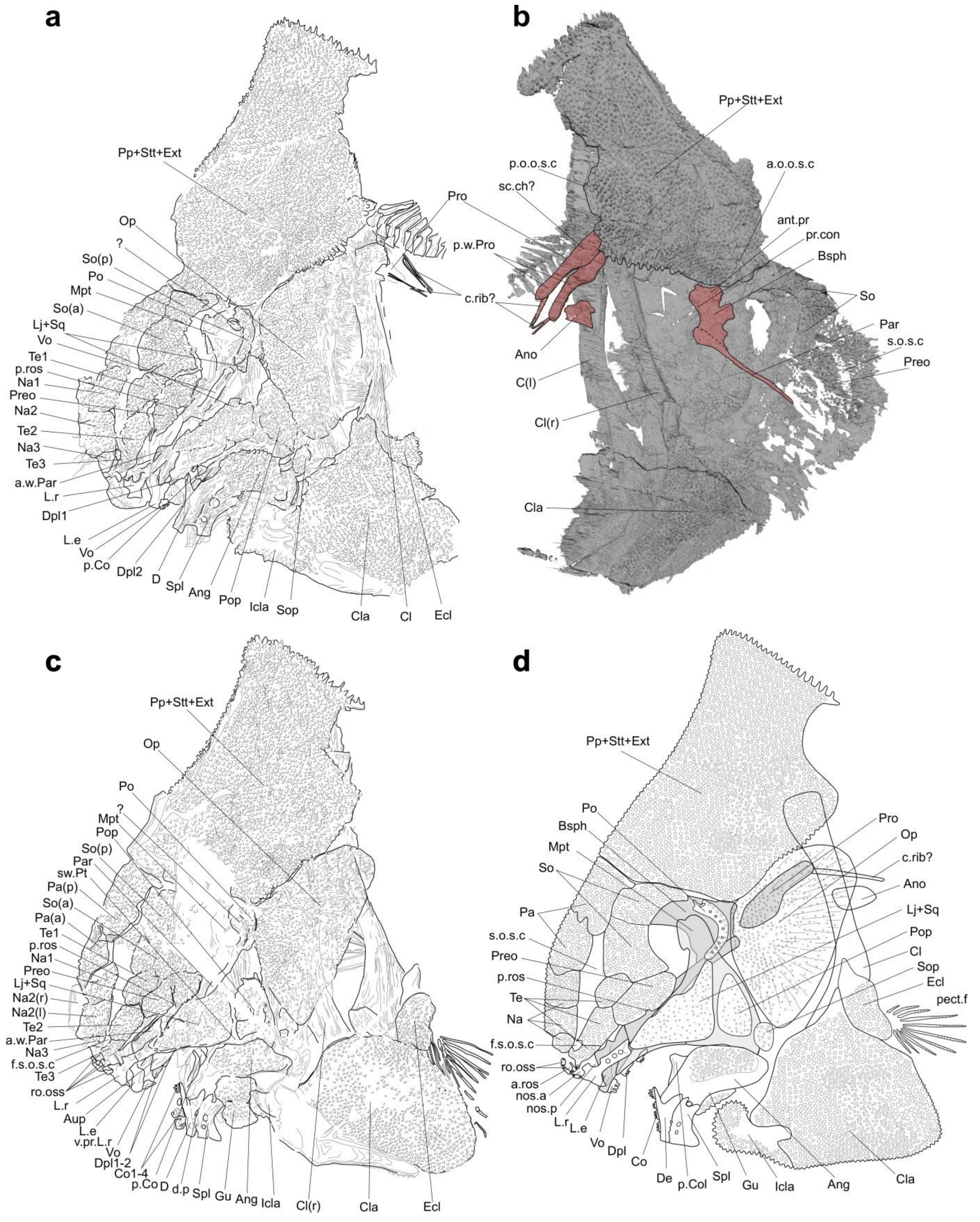

Figure S6. Skull of *Foreyia maxkuhni*, gen. et sp. nov., **a**, drawing of paratype (PIMUZ A/I 4372). **b**, surface CT image of the paratype (PIMUZ A/I 4372). **c**, drawing of holotype (PIMUZ A/I 4620). **d**, reconstruction. (a), anterior; Ang, angular; Ano, anocleithrum; a.o.o.s.c, anterior opening of the otic sensory canal; a.ros, opening of the anterior rostral organ; Aup, autopalatine; a.w.Par, anterior

wing of the parasphenoid; Bsph, basisphenoid; Cl, cleithrum; Cla, clavicle; Co, coronoids (numbered); c.rib?, possible cranial rib; De, dentary; d.p, enlarged sensory pore within the dentary; Dpl, dermopalatine (numbered); Ecl, extracleithrum; f.s.o.s.c, foramen of the supraorbital sensory canal; Gu, gular plate; Icla, interclavicle; L.e, lateral ethmoid; L.r, lateral rostral; Lj+Sq, lachrymojugal + squamosal; Mpt, metapterygoid; m.s.c, mandibular sensory canal; Na, nasal; nos.a, anterior nostril; nos.p, posterior nostril; Op, opercle; (p), posterior; Pa, parietal; Par, parasphenoid; p.Co, principal coronoid; pect.f, pectoral fin; Po, postorbital; Pop, preopercle; p.o.o.s.c, posterior opening of the otic sensory canal; Pp+Stt+Ext, ossification corresponding to the area occupied by the postorbital, supratemporal and extrascapulars in other actinistians; pr.con, processus connectens; Preo, preorbital; Pro, prootic; p.ros, posterior opening of the rostral organ; p.w.Pro, posterior wing of the prootic; ro.oss, rostral ossicles; sac.ch?, possible saccular chamber; So, supraorbital; Sop, subopercle; s.o.s.c, supraorbital sensory canal; Spl, splenial; sw.Pt, ventral swelling of the pterygoid; Te, tectal; (v), ventral; Vo, vomer.

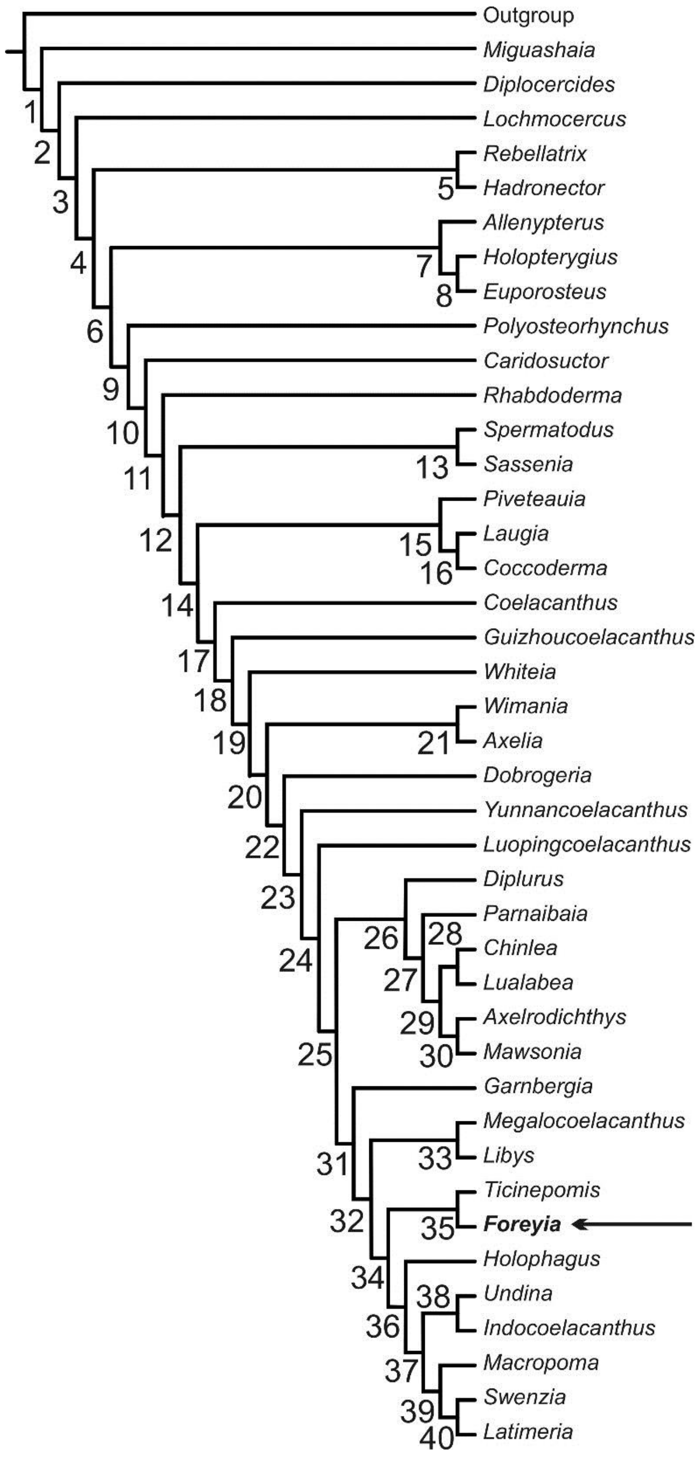

Figure S7. Cladistic analysis, one of the 259 most parsimonious tree. Tree length = 317, Consistency index = 0.3817, Homoplasy index = 0.6183, CI excluding uninformative characters = 0.3797, HI excluding uninformative characters = 0.6203, Retention index = 0.6766, Rescaled consistency index = 0.2582. Character changes (Character number, ci, character change, ->, ambiguous ; =>unambiguous ; character definitions are available in Supplementary Information): **Node 1:** 10, 0.250 0 -> 1; 33, 1.000 1 => 0; 71, 0.333 0 -> 1; 74, 0.500 0 -> 1; 83, 0.500 0 -> 1; 88, 1.000 0 => 1; 107, 0.200 0 -> 1; **Node 2:** 7, 0.333 0 => 1; 19, 1.000 0 => 1; 25, 0.200 0 -> 1; 93, 0.500 0 => 1; 95, 0.500 0 => 1; **Node 3:** 4, 0.500 0 -> 1; 23, 0.400 2 => 0; 24, 1.000 1 -> 0; 34,

0.500 1 -> 0; 45, 0.250 0 => 1; 55, 0.500 3 -> 0; 72, 1.000 1 -> 0; 90, 0.500 0 => 1; **Node 4:** 54, 0.500 0 -> 1; 94, 0.500 0 -> 1; **Node 5:** 23, 0.400 0 -> 1; 91, 0.250 0 -> 1; 102, 0.500 0 => 1; **Node 6:** 63, 0.333 0 => 1; 97, 0.250 0 => 1; **Node 7:** 8, 0.143 1 => 0; 25, 0.200 1 => 0; 29, 0.500 0 -> 1; 42, 0.250 0 -> 1; 48, 0.333 0 -> 1; 49, 0.400 1 -> 0; 50, 0.500 0 -> 1; 58, 0.500 0 -> 1; 94, 0.500 1 -> 0; 109, 1.000 0 => 1; **Node 8:** 15, 0.200 0 -> 1; 31, 0.333 1 -> 0; 32, 0.250 1 -> 0; 59, 0.167 1 -> 0; 93, 0.500 1 -> 0; 99, 0.250 0 -> 1; **Node 9:** 3, 0.250 1 -> 0; 11, 1.000 0 -> 1; 17, 0.400 0 -> 1; 55, 0.500 0 -> 4; 61, 1.000 0 => 1; 69, 0.500 0 -> 1; 73, 1.000 0 -> 1; 81, 1.000 0 -> 1; 83, 0.500 1 -> 0; 85, 1.000 0 -> 1; 87, 1.000 0 -> 1; 101, 0.333 0 => 1; **Node 10:** 1, 0.200 0 => 1; 20, 1.000 0 => 1; 56, 0.200 0 => 1; 59, 0.167 1 => 0; 67, 0.167 0 => 1; 102, 0.500 0 => 1; **Node 11:** 64, 0.333 0 => 1; ***Rhabdoderma*:** 8, 0.143 1 => 0; 46, 0.200 0 => 1; 63, 0.333 1 => 0; 77, 0.500 1 => 0; 78, 0.500 1 => 0; 97, 0.250 1 => 0; **Node 12:** 18, 0.250 0 -> 1; 29, 0.500 0 => 1; 62, 1.000 0 => 1; 101, 0.333 1 -> 0; 105, 0.333 0 -> 1; **Node 13:** 23, 0.400 0 => 2; 34, 0.500 0 => 1; 68, 0.400 0 -> 1; 80, 0.250 0 -> 1; 84, 0.333 0 -> 1; **Node 14:** 5, 0.250 0 => 1; 6, 0.500 0 -> 1; 44, 0.250 0 -> 1; 67, 0.167 1 => 0; 75, 1.000 0 => 1; **Node 15:** 32, 0.250 1 -> 0; 45, 0.250 1 => 0; 48, 0.333 0 => 1; 91, 0.250 0 => 1; 96, 0.286 0 -> 1; 100, 1.000 0 => 1; 108, 1.000 0 -> 1; **Node 16:** 18, 0.250 1 -> 0; 30, 0.200 1 => 0; 47, 0.250 0 => 1; 99, 0.250 0 => 1; **Node 17:** 1, 0.200 1 => 0; 21, 0.333 0 => 1; 27, 0.250 0 -> 1; 70, 0.250 1 -> 0; 71, 0.333 1 -> 0; 76, 1.000 0 -> 1; 77, 0.500 1 -> 0; 78, 0.500 1 -> 0; 82, 1.000 0 -> 1; 86, 1.000 0 -> 1; **Node 18:** 8, 0.143 1 => 0; 36, 0.200 0 => 1; 53, 0.500 0 -> 1; 96, 0.286 0 => 2; 97, 0.250 1 => 0; **Node 19:** 15, 0.200 0 => 1; 27, 0.250 1 -> 0; 59, 0.167 0 -> 1; 98, 0.333 0 => 1; **Node 20:** 17, 0.400 1 => 2; 26, 0.200 0 -> 1; 48, 0.333 0 -> 1; 79, 0.333 0 -> 1; 96, 0.286 2 -> 1; **Node 21:** 7, 0.333 1 => 0; 31, 0.333 1 -> 0; **Node 22:** 13, 0.500 0 => 1; 23, 0.400 0 -> 2; 35, 0.333 0 => 1; 104, 0.200 0 -> 1; **Node 23:** 32, 0.250 1 => 0; 59, 0.167 1 -> 0; **Node 24:** 8, 0.143 0 => 1; 10, 0.250 1 -> 0; 57, 0.333 0 => 1; **Node 25:** 27, 0.250 0 -> 1; 47, 0.250 0 -> 1; 52, 0.500 1 => 0; 91, 0.250 0 => 1; **Node 26:** 14, 0.500 1 => 0; 56, 0.200 1 => 0; 79, 0.333 1 -> 0; 92, 1.000 0 => 1; **Node 27:** 8, 0.143 1 -> 0; 15, 0.200 1 => 0; 17, 0.400 2 => 1; 43, 0.333 0 => 1; 45, 0.250 1 -> 0; 47, 0.250 1 -> 0; 65, 0.500 0 => 1; **Node 28:** 56, 0.200 0 -> 1; 67, 0.167 0 -> 1; 98, 0.333 1 -> 0; **Node 29:** 27, 0.250 1 => 2; 49, 0.400 1 => 2; 68, 0.400 0 -> 1; 106, 0.333 0 => 1; **Node 30:** 1, 0.200 0 => 1; 8, 0.143 0 -> 1; 16, 0.500 0 => 1; 30, 0.200 1 => 0; 36, 0.200 1 => 0; 38, 0.500 0 => 1; 41, 1.000 0 => 1; **Node 31:** 3, 0.250 0 -> 1; 22, 0.500 0 -> 1; 30, 0.200 1 -> 0; 60, 1.000 0 -> 1; 104, 0.200 1 => 0; 110, 1.000 0 -> 1; **Node 32:** 36, 0.200 1 => 0; 103, 0.500 0 => 1; **Node 33:** 2, 0.667 0 -> 1; 23, 0.400 2 -> 3; 39, 0.500 0 -> 1; 49, 0.400 1 -> 0; 50, 0.500 0 => 2; 68, 0.400 0 => 1; 70, 0.250 0 -> 1; 89, 0.333 0 -> 1; 99, 0.250 0 -> 1; **Node 34:** 27, 0.250 1 -> 0; 32, 0.250 0 => 1; 67, 0.167 0 => 1; 74, 0.500 1 -> 0; **Node 35:** 8, 0.143 1 => 0; 10, 0.250 0 -> 1; 18, 0.250 1 -> 0; 23, 0.400 2 -> 4; 35, 0.333 1 -> 0; 40, 0.500 0 -> 1; 42, 0.250 0 => 1; 43, 0.333 0 => 1; 44, 0.250 1 -> 0; 45, 0.250 1 -> 0; 105, 0.333 1 -> 0; 107, 0.200 1 => 0; **Node 36:** 9, 0.500 0 => 1; 64, 0.333 1 -> 0; **Node 37:** 28, 0.500 0 -> 1; 59, 0.167 0 => 1; 103, 0.500 1 => 0; **Node 38:** 5, 0.250 1 -> 0; **Node 39:** 39, 0.500 0 => 1; 40, 0.500 0 => 1; 51, 1.000 0 => 1; 64, 0.333 0 -> 1; 96, 0.286 1 => 2; **Node 40:** 23, 0.400 2 -> 0; 26, 0.200 1 => 0; 27, 0.250 0 -> 1; 30, 0.200 0 => 1; ***Undina*:** 63, 0.333 1 => 0; ***Diplocercides*:** 58, 0.500 0 => 1; 59, 0.167 1 => 0; 80, 0.250 0 -> 1; ***Coelacanthus*:** 4, 0.333 0 => 1; 10, 0.250 1 => 0; 26, 0.200 0 => 1; 37, 0.333 0 => 1; 42, 0.250 0 => 1; 47, 0.250 0 => 1; ***Whiteia*:** 5, 0.250 1 => 0; 6, 0.500 1 -> 0; 46, 0.200 0 => 1; 57, 0.333 0 => 1; 84, 0.333 0 => 1; 107, 10.200 1 => 0; ***Chinlea*:** 65, 0.500 1 => 0; ***Axelrodichthys*:** 37, 0.333 0 => 1; ***Parnaibaia*:** 3, 0.250 0 => 1; 4, 0.333 0 => 1; ***Diplurus*:** 9, 0.500 0 => 1; 23, 0.400 2 -> 0; 36, 0.200 1 => 0; 42, 0.250 0 => 1; 44, 0.250 1 -> 0; 46, 0.200 0 => 1; 49,

0.400 1 => 0; 50, 0.500 0 => 1; 57, 0.333 1 => 0; 107, 0.200 1 => 0; ***Holophagus*****:** 1, 0.200 0 => 1; 99, 0.250 0 => 1; ***Macropoma*****:** 2, 0.667 0 => 2; 28, 0.500 1 -> 0; 67, 0.167 1 => 0; 89, 0.333 0 => 1; 104, 0.200 0 => 1; ***Latimeria*****:** 4, 0.333 0 => 1; 27, 0.250 1 -> 2; 46, 0.200 0 => 1; 50, 0.500 0 => 1; 107, 0.200 1 => 0; *Swenzia*: 2, 0.667 0 => 1; ***Ticinepomis*****:** 55, 0.500 4 => 3; ***Foreyia*****:** 96, 0.286 1 => 0; 104, 0.200 0 => 1; ***Megalocoelacanthus*****:** 5, 0.250 1 => 0; 56, 0.200 1 => 0; ***Garnbergia*****:** 98, 0.333 1 => 0; ***Luopingcoelacantus*****:** 35, 0.333 1 => 0; 96, 0.286 1 => 0; ***Yunnancoelacanthus*****:** 15, 0.200 1 => 0; 16, 0.500 0 => 1; 17, 0.400 2 => 0; 25, 0.200 1 => 0; 36, 0.200 1 => 0; 54, 0.500 1 => 0; 68, 0.400 0 => 2; ***Dobrogeria*****:** 1, 0.200 0 => 1; 71, 0.333 0 -> 1; ***Axelia*****:** 68, 0.400 0 => 1; ***Guizhoucoelacantus*****:** 17, 0.400 1 => 0; 25, 0.200 1 => 0; 43, 0.333 0 => 1; 44, 0.250 1 -> 0; 52, 0.500 1 => 0; 95, 0.500 1 => 0; 105, 0.333 1 -> 0; 106, 0.333 0 => 1; ***Laugia*****:** 7, 0.333 1 => 0; 26, 0.200 0 => 1; 31, 0.333 1 => 0; 56, 0.200 1 => 0; ***Coccoderma*****:** 23, 0.400 0 => 2; 27, 0.250 0 => 1; 37, 0.333 0 => 1; 38, 0.500 0 => 1; 49, 0.400 1 => 0; 89, 0.333 0 => 1; 96, 0.286 1 -> 0; 97, 0.250 1 => 0; 106, 0.333 0 => 1; ***Piveteauia*****:** 13, 0.500 0 => 1; 22, 0.500 0 => 1; 46, 0.200 0 => 1; 53, 0.500 0 => 1; 79, 0.333 0 => 1; ***Spermatodus*****:** 15, 0.200 0 => 1; 26, 0.200 0 => 1; 70, 0.250 1 => 0; ***Sassenia*****:** 18, 0.250 1 -> 0; 69, 0.500 1 => 0; ***Polyosteorhynchus*****:** 21, 0.333 0 => 1; 23, 0.400 0 => 1; 91, 0.250 0 => 1; ***Holopterygius*****:** 70, 0.250 1 => 0; ***Euporosteus*****:** 80, 0.250 0 -> 1; 104, 0.200 0 => 1; ***Miguashaia*****:** 12, 0.500 0 -> 1; 66, 1.000 0 => 1: ***Lochmocercus*****:** 21, 0.333 0 => 1; 101, 0.333 0 => 1.

B – Geological setting (Fig. S1)

The up to 3000 m high mountains of the Ducan area southwest of Davos (Eastern Swiss Alps, Canton Graubünden) are built by strongly deformed series of Triassic and Permian sediments of the Austroalpine Silvretta Nappe[1,2]. Embedded in light Middle Triassic shallow water carbonates, the Prosanto Formation comprises a sequence of dark limestones, shales and dolomites measuring about 120 m in thickness. Its diverse and well-preserved actinopterygian fish fauna suggest a deposition in stagnant abiotic, probably anoxic bottom water conditions in a small intraplatform basin[3]. Small plankton feeding and larger predatory fishes, together with sauropterygian reptiles probably lived in the surface water. Medium sized fishes feeding on hard-shelled bivalves, crustaceans, and calcareous algae must have lived at the border of the basin in a shallow water environment. Terrestrial plants, a few insects, a rauisuchian and a protorosaurian reptile[4-7] were probably washed in by storms. Lithostratigraphy and fossils share many similarities with the classic Middle Triassic fossil site of Monte San Giorgio area in the southern Alps (Anisian/Ladinian), corroborated by U/Pb zircon ages of 240.91 ± 0.26 Ma from a volcanic ash layer in the fossiliferous beds of the upper Prosanto Formation and the overlying Altein Formation (239.89 ± 0.21 Ma)[8]. That suggests a correlation of the upper Prosanto Formation with the lower Meride Limestone (*P. gredleri* Zone, Early Ladinian[8-10]). In 2013, Cavin et al.[11] described the first two coelacanths, found in the middle and upper part of the Prosanto Formation as *Ticinepomis* cf. *T. peyeri*.

C – Osteological description of *Foreyia maxkuhni* gen. et sp. nov. (Figs S2-7)

Dermal skull roof

All the bones of the skull roof of the parietonasal and postparietal shields are ornamented with strong tubercles, which are regularly spaced and rather homogenous in size (Fig. 2C1). On both specimens, a series of tall blunt spine-like tubercles are aligned along the outline margin of the postparietal shield (Fig. 2C2). CT scan of the paratype shows that spines from both sides were arranged side by side but alternate between both sides. On the paratype only, tubercles on the posterolateral corner of the postparietal shield are taller than on the rest of the skull roof. Because of the strong ornamentation, paths of the sensory system are hard to detect. Odontodes very similar in shape and structure were studied by Ørvig (1977)[12] on a related form from the Middle Triassic of Monte San Giorgio, whose anatomical description is pending.

The skull roofing bones of the parietonasal shield (roofing the ethmosphenoid portion of the braincase) comprise five paired ossifications forming the mediolateral series along the midline. The posterior two pairs are the posterior and anterior parietals (**Pa**) and the anterior three pairs are the nasals (**Na**). The width of the parietals and of the posterior nasals is constant and their length is only slightly decreasing from the posterior parietals to the posterior nasals, with the posterior parietal being 1.5 longer than the posterior nasal. The quadrangular anterior-most nasals are significantly smaller than the other bones of the series. The sutures between both pairs of nasals and between the posterior nasal and the anterior parietal are simple. The anterior parietal partially overlaps the posterior parietal and shows an interdigitate suture, as in *Latimeria*[13]. Lateral to the parietal and nasal series is a lateral series of five bones corresponding to two supraorbitals (**So**) posteriorly (above the orbit) and three tectals (**Te**) anteriorly (roofing laterally the ethmoid region). The posterior supraorbital is a large ossification with a posterolateral process. The anterior supraorbital is the largest bone of the series, quadrangular in shape with a curved orbital margin. On both specimens, between the mediolateral and the lateral series runs a wide groove with irregular margins and devoid of tubercles on its bottom. It likely accommodated the supraorbital sensory canal (**s.o.s.c**). The groove tapers anteriorly. An oval foramen opens just in front of the groove, at the level of the dorsal contact between the first and second tectal (**f.s.o.s.c**) (Fig. S4). The anteriormost tectal bears a little anteroventral extension, devoid of ornamentation, which ended by a small pore corresponding to the connection of the supraorbital sensory canal with the infraorbital sensory canal. Several tiny loosely connected bones, which form the tip of the snout in the holotype, are rostral ossicles (**ros.oss**) (Fig. S4). One of these is a three-branched radiating ossification, which makes the connection between the supraorbital canal and the ethmoid commissure, and possibly with the infraorbital sensory canal as well. Anteriorly are located two tiny tube-like rostral ossicles, which housed the ethmoid commissure. From the tip of the snout, the infraorbital sensory canal runs posteriorly through the lateral rostral (**L.r**). It is a large bone formed by an expanded anterior portion with a dorsal process carrying the connection to the supraorbital canal, with a ventral expansion contacting the lateral ethmoid and eventually with an elongated posterior shaft with parallel margin. The posterior shaft is crushed in both specimens, but three rounded openings for the sensory canal are still visible. Posteriorly, the sensory canal exits through a large opening. Based on comparison with *Latimeria*[13], we consider that the space delimitated by the three rostral ossicles corresponds to the opening for the anterior rostral organ (**a.ros**), while a concavity dug in the dorsal margin and one dug in the

anterodorsal margins of the lateral rostral correspond to the posterior (**nos.p**) and anterior nostrils (**nos.a**), respectively, while the opening for the posterior rostral organ opens in the preorbital (see below) (Fig. S6d). On the paratype, a thin bony plate bearing spaced out tubercles is present at the very tip of the snout. Its localization indicates that it might be a premaxilla, which forms a hemispherical premaxillary-rostral cap in some coelacanths such as *Macropoma*[13]. But its thin plate-like structure and its general shape suggest that it is a shifted bone of the cheek, possibly an upside-downed lachrymojugal-squamosal from the right face of the specimen. Wedged between the anterior supraorbital and both posterior tectals is the preorbital (**Preo**). It is an elongated ovoid bone with a notch in the mid-length of the ventral margin of the bone. The notch marks a short groove leading to an opening, which corresponds to the posterior opening of the rostral organ (**p.ros**). There are usually two posterior openings for the rostral organs in actinistians, which may open between distinct ossifications (e.g. *Latimeria*) or within the preorbital only (e.g. *Allenypterus, Rhabdoderma, Hadronector,* etc.). Both openings can merge at the surface of the bone (e.g. *Diplocercides*), which would correspond to the situation present in *Foreyia*.

The postparietal shield, which roofs the otico-occipital portion of the braincase, is proportionally the broadest element of the skull. It somehow mirrors the hypertrophied clavicle located at the posteroventral corner of the pectoral girdle. The posterior margin of the shield forms a perpendicular line to the long axis of the skull, but because of the lateral flattering of the head and of the body, the occiput region probably formed on the living fish a pyramidal dome overhanging the head. No limits between ossifications are visible in the postparietal shield, neither with optical instruments nor with CT images (Fig. S5; Smovie). Breaks and grooves are present on both specimens, but they appear to be the result of the crushing of the skull roof before fossilization. We hypothesize that the postparietal shield is composed of a single, paired or unpaired, ossification resulting either from the complete fusion of original ossifications (postparietals, supratemporals and extrascapulars), or by the topographic invasion of one or more bones of the whole postparietal shield (**Pp+Stt+Ext**). The lateral contour of the postparietal shield forms a straight margin along the dorsal margin of the opercle, then turns at right angle and draws a concavity. In the holotype, fragments of laminar bones without ornamentation, corresponding either to deeper portions of the superficial dermal bones or to endochondral occipital ossifications, are visible in the concavity. The posterior margin, which bears the above described large tubercles, extends posteriorly slightly over the occiput. On both specimens, a split is present between this posterior band and the main body of the shield. But this mark probably corresponds to a breakage associated with the flattening of the specimen rather than to a suture between ossifications. Because of the strong ornamentation of the postparietal shield, grooves for pit lines and pores corresponding to the paths of the sensory canal are hard to detect. On the paratype, the anterior entry of the otic sensory canal (**a.o.o.s.c**) is located on the anterior margin of the rounded anterolateral corner of the shield. On both specimens, the posterior opening of the otic sensory canal (**p.o.o.s.c**) is located at the posterolateral corner of the shield, just above the level of the neural arch of the vertebrae. The CT images of the paratype do not allow following the sensory canals within the bones, except along a few millimeters near the posterior exit of the otic canal. The anterior margin of the postparietal shield, which contacts the posterior border of the ethmosphenoid portion of the skull roof, is smooth and slightly undulating.

Notwithstanding its relative short length, the postparietal shield of *Foreyia* is derived and hardly comparable to the condition observed in the other coelacanths because of the fusion (or topographic invasion) of the original bones forming the shield. A fusion of the postparietal with the supratemporal occurs in *Ticinepomis* (see below). Inclusion of extrascapular ossifications within the postparietal shield is present in several mawsoniid genera, such as *Trachymetopon* (Dutel et al., 2015), *Mawsonia* and *Axelrodichthys* (Maisey, 1986), but the pattern in *Foreyia* cannot be compared with those of mawsoniids.

Cheek bones and opercle

The cheek bones of *Foreyia* are ornamented with tubercles smaller than on the skull roof. The ossifications are very thin and their outlines are not easily recognizable. The ossifications are separated from each other by gaps. A probable lachrymojugal-squamosal, a postorbital, a preopercle and a probable subopercle are present. No spiracular has been observed. The postorbital (**Po**) is a curved bone along the posteroventral corner of the orbit, with almost parallel margins. The dorsal part, as preserved on the paratype, shows two poorly preserved indentations along its dorsal margin. The anterior indentation faces a process with a rough surface on the posterolateral corner of the posterior supraorbital. In the latimeriids *Macropoma*, *Swenzia* and *Latimeria*, a similar excavation located in the anterodorsal corner of the postorbital is present and receives a tough ligamentous connection with the posterior supraorbital. The posterior indentation corresponds to the exit of the infraorbital sensory canal, which reaches the anterior opening of the otic sensory canal. The postorbital bears strong spiny tubercles, especially along its ventral branch, which also shows openings for the infraorbital canal. The bone located anteriorly to the postorbital has a shape unique among coelacanths. It is a large triangular plate-like ossification with blunt angles. The longest border is the dorsal one, which is aligned with the parasphenoid. The bone extends ventrally and covers the cheek, with an anteroventral margin running along the border of the pterygopalatine and a posteroventral margin along the preopercle. Based on the shape and size of this ossification, we consider that it corresponds to the fusion of the lachrymojugal and squamosal (**Lj+Sq**). In both specimens, this ossification appears to be composed of two elements separated by a vertical arched gap almost in its mid-length, but this break is probably caused by an underlying ridge corresponding to the suture of the autopalatine with the pterygoid located underneath. The extremity of the jugular canal is visible on the paratype and is aligned with the entry of the canal in the preopercle posteriorly. The preopercle (**Pop**) is a roughly triangular (holotype) or ovoid (paratype) ossification located along the ventral part of the anterior margin of the opercle (**Op**). The entry of the jugular sensory canal (**j.s.c**) is marked by a groove perpendicular to the anterior margin situated just below (holotype) or above (paratype) the mid-depth of the bone. A small ovoid subopercle (**Sop**) is identified thanks to a small patch of tubercle wedged between the ventral extremities of the opercle and preopercle, and rests on the lateral side of the articular head of the quadrate. The opercle is short and deep, somehow more developed in the paratype than in the holotype. It completely covers a section of the vertical limb of the cleithrum, which is a feature unique among actinistians. Indeed, coelacanths usually have the cleithrum located posteriorly to the opercle. The shape of the opercle is intermediate between a triangular and an ovoid form, with its anterior corner forming an open

rounded angle. It is covered with tubercles intermediate in size between the large tubercles of the dermal bones of the skull roof, and the small tubercles of the cheek bone. Tubercles are mostly concentrated in the anterior and central parts, while the posterior margin is ornamented with fine radiating ridges.

Lower jaw

The lower jaw of *Foreyia* is highly derived but the typical actinistian organization of the mandible is recognized. The general shape of the lower jaw is comma-shaped with its dorsal contour forming almost a half-circle. Because of the mode of preservation of both specimens, only the lateral ossifications of the lower jaw are visible. The splenial (**Spl**) has a deep and straight symphyseal margin and a concave ventral margin. In the paratype, the splenial has a well-marked reticulated ornamentation, which is absent in the holotype. The anteroventral corner has a rounded margin which forms a well-marked ‘chin’. The posterior margin of the splenial is notched to form the anterior border of a large sensory pore. Two other pores open in the mid-depth of the bone in its anterior half. The dentary (**De**) is short and deep and its surface is almost smooth in both specimens. Its ventral margin contacts the splenial along a curved suture. Close to this suture is an enlarged sensory pore (**d.p**), which is a synapomorphy of the inclusive clade encompassing *Polyosteorhynchus* and *Latimera* (Fig. 2a). The oral border is straight and extends posteriorly as an elongated and thin process. This process forms the upper limb of a deep notch on the posterior margin of the bone. The process is homologous with the hooked-shaped process present in *Latimeria* and some extinct coelacanths, which received the maxillary fold of skin from the upper jaw. If the size of the notch is somehow proportional to the size of the maxillary fold, the latter should have been large in *Foreyia*. Four slightly displaced coronoids (**Co**) are visible along the dorsal margin of the dentary of the holotype (Figs 2C7, S4). They were originally borne by bones from the medial side of the mandible (mentomeckelian, prearticular). The two anterior coronoids are roughly rectangular and their tooth-bearing surface is concave. Each bears between 8 and 10 conical teeth of similar size with a blunt apex. The third coronoid is visible as a small shifted piece of bone but we cannot identify any teeth. The fourth coronoid is located at the tip of the dorsal process of the dentary. Four teeth are visible in lateral view, with one being slightly larger than the others. The principal coronoid (**p.Co**) is better visible on the holotype, where it is slightly shifted. It is formed by an anterior limb with a shallow ridge and by a posterior broad plate. This plate is thin, except along its anterior margin, which forms a strong ridge. The ridge marks the contour of the adductor muscle by comparison with *Latimeria*[13]. The angular (**Ang**) is comma-shaped and partly covered with the strong ornamentation present on the skull roof and more pronounced in the paratype than in the holotype. The anterior and ventral areas are devoid of ornamentation, and the limit between ornamented and smooth region parallel to the ventral margin forms a ridge, under which ran the mandibular sensory canal (**m.s.c**) as indicates a foramen visible on the paratype. Another opening, close to the limit with the splenial, is visible in the paratype. The posterior margin of the lower jaw is rounded. The lateral surface bears no tubercles but is pitted. On the paratype, a shallow groove indicates the suture with the retroarticular (**Rart**), which is hardly visible in lateral view. The mandible is very derived and hardly comparable at first sight with the mandible of other coelacanths.

In *Allenypterus*, the lower jaw is also deep, short and curved, with a rather deep symphysis. However, the dentary has a simple quadrangular shape contrary to the hooked-shape dentary of *Foreyia*, which is a character diagnostic of the more derived coelacanths[13]. A butterfly-like rounded ossification is present under the lower jaw of the holotype. It bears a strong ornamentation made of the same-sized tubercles as on the skull roof and shows anteriorly a notch extending through the mid-line as a groove. We regard this ossification as the pair of gular (**Gu**) plates present in other coelacanths, which are here partly fused together.

Neurocranium, parasphenoid and vomer

The lateral ethmoid, the parasphenoid, the vomer and, thanks to the CT images, part of the basisphenoid and of the prootics are visible to some extent. The *processus connectens* (**pr.con**) of the basisphenoid (**Bsph**) from the right side of the paratype can be distinguised on the CT images (Figs 2B, S5, Smovie). On the left side of both specimens, a protruding process, dug with a concavity and extended dorsally with a crest, is present at the level of the internal curvature of the postorbital (labelled with '?' on the Fig. 2b). We cannot decide if the concavity corresponds to a suprapterygoid fossa with the upper ridge forming the base of the antotic process, or if the whole process is a large basipterygoid process, only present in basal coelacanths. Because of the relatively high position of this process on the basisphenoid, we favour the first hypothesis. The angle formed between the axis of the processus connectens and the axis of the parasphenoid is very open, about 160°. The posteroventral corner of the ethmosphenoid block, apparently formed by the parasphenoid, protrudes posteriorly. The lateral ethmoid (**L.e**) forms anteriorly two parts arranged in an open angle for the nasal capsule. The posterior part of the bone is triangular and broadens posteriorly. Its dorsal margin is thickens and delimitates ventrally the ventrolateral fossa (**v.l.fo**), which receives the autopalatine. The anterior quadrangular part has its ventral margin aligned with the roof of the mouth and bears a swelling at its posteroventral corner, which articulates with the ventral process of the lateral rostral (**v.pr.L.r**). In basal coelacanths, the ethmoid region is strongly ossified and bears a deep ventrolateral fossa, but the lateral ethmoids are not individualized with a distinct suture (*Diplocercides*, *Euporosteus*). In most other genera where the ethmoid region of the neurocranium is known (*Latimeria, Rhabdoderma*, *Laugia*), the lateral ethmoids are generally small ossifications only partially ossified, which passes into cartilage forming the floor of the nasal capsules. But in all these instances, the lateral ethmoids are always proportionally much shallower than in *Foreyia*. A small vomer (**Vo**), with five small conical blunt teeth in both specimens (but more may have been present) is located just below the anterior part of the lateral ethmoid. The parasphenoid (**Par**) apparently extends posteriorly back to the tip of the basisphenoid block but it seems that it has no contact with the processus connectens. Its shaft is slightly shifted on both specimen and shows a concave edentulous ventral side just anterior to the orbit. We cannot see, however, if a tooth patch is present more anteriorly. An anterior wing (**a.w.Par**) rises dorsally and seems to extend well backwards. CT images of the paratype show, posteriorly to the skull roof and fully embedded in the matrix, two rounded processes, which extend posteriorly from the postparital shield and which overpass posteriorly the cleithra. The anterior parts of these elements are slightly swollen. Our interpretation is that they correspond to the prootics (**Pro**), represented mostly by the posterior wings (**p.w.Pro**) and

possibly by the saccular chambers (**sac.ch?**) anteriorly, which have been shifted posteriorly during the crushing of the skeleton before fossilization. The wings are associated with rod-like elements visible externally on the paratype. Several thin rods are present on each side, but they may correspond to a pair of single elements with a groove running along their length, which are now fragmented. The left rod-like bone rests on the external side of the left wing and the right rod-like bone rests on the internal side of the right wing. The interpretation of these elements is difficult. We suggest that they might be cranial ribs (**c.rib?**) as observed in Recent and some extinct lungfishes. To our knowledge, cranial ribs have never been described in actinistians. Moreover, when present, cranial ribs in lungfishes are associated with occipital ossifications while these elements are associated here with otic ossifications. The function of cranial ribs in lungfishes is still debated. Their presence was regarded as evidence of air-breathing[14-18] or was regarded as associated to suctorial actions of the jaws in order to assist feeding[19,20]. In coelacanths, the presence of a lung is likely a primitive character. In some coelacanths, including *Latimeria*, bony plates cover the lung and may have helped to improve lung ventilation during air breathing[21-23]. Both specimens of *Foreyia* show no evidence of a calcified lung, and the possible cranial ribs in this coelacanth, if confirmed in future, are probably not associated with air breathing.

Palatoquadrate

The general shape of the palatoquadrate is visible but details are difficult to appreciate because of the cheek bones covering it. The general shape is triangular as in other coelacanths, but because of the general head shape, it is distinguishable from any other genera by being deeper than long. The autopalatine (**Aup**) is visible on both specimens as a triangular bone that fits anteriorly in the ventrolateral fossa of the lateral ethmoid. It is also unique among coelacanths by its proportionally large size occupying almost half of the length of the pterygopalatine. Two small dermopalatines (**Dpl**), each bearing few teeth similar in size are present along the oral margin of the autopalatine. The metapterygoid (**Mpt**) contour is hardly identifiable, but it is very deep as indicates its extension visible in the orbit of the paratype (Fig. S3). The quadrate is only partly visible. The pterygoid is visible in the gap between the ossifications of the cheek. Close to its posteroventral extremity, the oral margin of the pterygoid forms a swelling (**sw.Pt**) as observed in latimeriids[24], but shallower in *Foreyia*. The anterior part of the palatoquadrate is deep compared to most other coelacanth genera. It has also been described as deep in *Ticinepomis*[25].

Axial skeleton

The postcranial skeleton of *Foreyia* is derived compared to the postcranial skeleton of other coelacanths, but typical actinistian characters are recognized (Figs 1, S2,3). The vertebral column is very short with 17 abdominal and 18 caudal vertebrae. This number is the lowest known for coelacanths caused by the low amount of abdominal vertebrae. The neural spines (**n.s**) increase in size backwards along the first six vertebrae. Abdominal neural spines have parallel margin and caudal neural spines have an enlarged distal extremity. The first four haemal spines (**h.s**) are

relatively narrow and long, and do not support the caudal fin. The next 14 haemal spines, which support the ventral lobe of the caudal fin, have expanded distal extremities. The 14 ventral and 16 dorsal radials (**Ra**) are symmetrical flatten rod of bones with proximal and distal expanded extremities.

Pectoral girdle and fin

The cleithrum (**Cl**) is visible on both specimens. The dorsal half of the bone is proportionally narrow with parallel margins but its dorsal extremity, hidden under the hypertrophied post-parietal shield, appears to broaden slightly. The CT images show the dorsal extremities of the cleithra applied against the postparietal shield, but the exact nature of the connection between the pectoral girdle and the skull is not understood. Anocleithra (**Ano**) are not visible externally, but the CT images reveal in the matrix a paired ossifications oriented posteriorly located on the internal side of the cleithra in the mid-depth of their vertical branch. These bones are regarded as modified anocleithra, which have an unusual location and orientation compared to other coelacanths. The ventral half of the cleithrum broadens, with its anterior margin forming a regular curve. Its ventral extremity is hidden under the hypertrophied clavicle (**Cla**). The latter ossification forms a large triangular plate with a huge expending ventroposterior expansion and a sinusoidal anterior margin. The ventral margin is almost straight. Although this part of the pectoral girdle is damage on both specimens, the paratype shows that both cleithra were fused along the midline. The ventral side of the paired cleithra probably formed a flat area on the living fish, which explain why the ossifications are broken on both specimens under the pressure of the sediment. The clavicle is almost completely covered with the same strong ornamentation as present on the skull roof. The dorsal-most tip of the clavicle, which is tightly attached to the cleithrum, forms a pointed process devoid of tubercles. A reniform extracleithrum (**Ecl**), an ossification unique for coelacanths, lined a concavity of the posterodorsal corner of the clavicle. It is covered by the same kind of tubercles, excepted an unornamented pointed process dorsally, which fits in a groove along the cleithrum. Fused to the anteroventral corners of both clavicles is an unpaired bone, which is regarded as an interclavicle (**Icl**). This ossification is present in both specimens, but better preserved on the paratype. The bone is composed of a thick and roughly circular posterior part, which contacts the clavicle posteriorly through a V-shaped suture. The anterior part expends as a hemispherical structure, which lies very close to the concave ventral side of the mandible. The ventral side and the hemispherical part are covered with large tubercles. Alternatively, this bone might be a much modified urohyal, but its strong ornamentation and its suture with the clavicle make this hypothesis less likely. The pectoral fin (**pect.f**) is reduced in size and composed of 10 rays. Among coelacanths, *Allenypterus* only has less pectoral fin rays (9). The rays are segmented and unbranched.

The shoulder girdle of coelacanths was said to be remarkably conservative, except in *Miguashaia*[13]. *Foreyia* is another exception, and it shows some similarities with *Miguashaia*. The occurrence in *Foreyia* of an extracleithrum is a synapomorphy of coelacanths. Its large ovoid shape is more reminiscent of the extracleithrum of the basal *Miguashaia* than that of the more derived genera, in which it is much more slender[26,27]. The vertical limb of the cleithrum is very elongated in *Foreyia*,

due to deepening of the skull, but its shape is otherwise typical for coelacanths. In the other members of the clade, however, the pectoral girdle lies free from the skull, but in *Foreyia*, the dorsal tip of the cleithrum is located close to the occipital region of the braincase and the anocleithrum has an unusual low position in the girdle. We cannot detect, however, if a bony contact exists between the skull and the pectoral girdle because this part of the skull is hidden. It is likely, however, that there is a structural connection between both entities and that the spatial proximity between them is due to the highly modified posterior part of the skull. The clavicle, also, has the usual position in coelacanth pectoral girdle, i.e. overlapping the ventral part of the cleithrum, but its extreme development makes it unique among the clade. In *Miguashaia*, the clavicle is proportionally large, although not in the same proportions, but it lies in a more anterior position[26,27]. Most coelacanths have no interclavicle, with the exception of *Whitheia* and *Laugia*, in which it is a small subdermal ossification of probable endochondral origin[13] and *Miguashaia*, in which the bone bears ornamentation and has a dermal origin[27]. Forey *et al*. (2000)[27] did not figure the interclavicle in *Miguashaia*, but they stated that it is very similar to the interclavicle figured by Jarvik (1972)[28] and referred to *Glyptolepis* sp. The interclavicle of *Foreyia* shares with the interclavicle figured by Jarvik[28] the rounded anterior extremity and the presence of ornamentation on the ventral and ventrolateral sides, but this ornamentation is much more developed in *Foreyia*.

Pelvic girdle and fin

Nothing is preserved of the pelvic girdle but both pelvic fins (**pelv.f**) are visible on the holotype. Twelve rays are present, which is a low amount compared to other coelacanths (*Allenypterus* has 6 and *Hadronector* has also 12 rays). The rays are segmented and unbranched, and bears denticles especially well-developed in the anterior-most rays.

Unpaired fins

None of the basal plates (anterior and posterior dorsal basal plates, anal basal plate) are preserved on the available specimens. All the fin rays are segmented and unbranched, as in most coelacanths. Denticles are present on the fin rays of the anterior dorsal (Fig. 2C3) and of caudal fins (as well as on the pelvic fins), but not on the posterior dorsal fin (and on the pectoral fins). They are relatively well-developed on the anterior dorsal fin and on the anterior most caudal fin rays, but minute on most of the caudal fin rays. Both dorsal fins (**dor.f**) and the caudal fin (**cau.f**) are very large compared to the body size. All the fin rays are unbranched as it is the case in almost all coelacanths. The anterior dorsal fin has 15 rays, the maximum of rays observed in coelacanths together with *Allenypterus*, and the posterior dorsal fin has 17 rays, a number situated within the range observed in the coelacanths. The caudal fin shows a one-to-one ratio between the radials and the fin rays. The supplementary caudal fin lobe (**sup.cau.f.l**) is supported by approximately eight dorsal and eight ventral rays. It is well-developed although it barely protrudes the caudal fins contour posteriorly because the dorsal and ventral lobes are very large.

Scales

The scales show variations according to their position on the body (Fig. 2C,D). The common features that all scales share are the approximately circular exposed shape and the occurrence of two to four spines aligned on an anteroposterior axis. The ventral-most scales located on the belly, between the hypertrophied clavicle and the pelvic fins appear to be very thick, without superficial ornament and with two to four spines, or denticles. There is generally one well-developed spine accompanied by one to three much smaller spines. It seems that the whole arear of the scales is exposed to the surface, although we cannot check this feature with certainty. These scales form a paving-like structure, which may have acted as an armoured protection on the belly. Higher on the flank, the scales become thinner, the overlapping between scales increases and the spines are proportionally smaller. There is still one more developed spine close to the posterior margin preceded anteriorly by one or two smaller spines. The spines decrease in size towards to anterior portion of the flank or are even absent. The exposed surface is ornamented with fine ridges diverging from the spines. The posterior margin of the scales bears between 5 and 10 fine denticulations. In coelacanths, scales are generally largely overlapping with only about one third of the scales exposed. In *Foreyia*, however, the overlapping between scales seems to be less pronounced, especially in the ventral area.

D – Relationships, character definitions and datamatrix for phylogenetic analysis

Relationships

At first sight, the general head morphology of *Foreyia* is reminiscent to the head morphology of *Allenypterus*, in particular the steep and convex profile of the anterior moiety in lateral view, and the proportionally short and deep mandible. However, closer examination shows striking differences between both genera, in particular in the posterior moiety of the skull roof (although both genera share an almost equidimensional postparietal, which is an unusual feature in coelacanths[13]), in the cheek bones and, obviously, in the postcranial features.

In the course of this study, we paid a special attention to *Ticinepomis*, a genus recovered nearby the locality of *Foreyia*, but in the middle part of the same formation. The holotype of *T. peyeri* was described from the Middle Triassic of Monte San Giorgio by Rieppel[25] in 1980, then revised[11]. Although being very different from *Foreyia*, both taxa share some features not included in the cladistic analysis. Most of the shared characters are less pronounced in *Ticinepomis*, but they herald the extreme development observed in *Foreyia*.

The phylogenetic relationships of *Ticinepomis* have been much discussed. In 1980, Rieppel[25] pointed out several characters of *Ticinepomis* that he regarded as primitive for actinistians, such as the plate-like premaxilla and a distinct horizontal portion of the clavicle. In 1991, Cloutier[29] resolved *Ticinepomis* as the basal-most member of a clade including *Coelacanthus*, *Axelia* and *Wimania*. Forey[13] excluded *Ticinepomis* from his cladistic analysis because this genus raised instability in the analysis. Dutel et al.[24] found *Ticinepomis* as the basal-most latimeriid and Cavin et al.[11] and Cavin &

Gradinaru[30] found *Ticinepomis* deeply nested within the latimeriids. As pointed out by Forey[13], the instability brought by *Ticinepomis* in the analysis of actinistians is not due to a lack of data, but to contradictions in the distribution of characters.

During the process of completing the datamatrix of Cavin & Gradinaru[30], we corrected character states for *Ticinepomis* based on new observations (characters [8], [12], [27], [28], [33], [37], [40], [52], [75]). When character states differed between *Ticinepomis peyeri* from Monte San Giorgio and *Ticinepomis* cf. *T. peyeri* from the Prosanto Formation (Character [57], [62]), we coded the character as polymorphic.

In order to resolve the phylogenetic relationships of *Foreyia* and *Ticinepomis* among the coelacanths, we performed a cladistic analysis. We used Cavin & Gradinaru's datamatrix[30], which is based on Forey's datamatrix[13] with the inclusion of several taxa described since, as well as some corrections of coding made by various authors. Moreover, we redefine here a few characters and corrected some of the previous coding.

Macropoma has a premaxillary-rostral complex with teeth at its surface[13], as well as *Swenzia*[31]. In *Laugia*, also, a similar hemispherical complex is present with teeth located along the oral margin only and perforated by pores for the ethmoid commissure. Forey[13] is uncertain about the original interpretation of the structure in *Laugia* by Stensiö[32], and suggested that the complex may correspond to a series of small rostrals, although he coded the situation as derived, i.e. 'snout bones consolidated' in his datamatrix. The latimeriid *Megalocoelacanthus* also has a consolidated snout bone, but no teeth are present[24]. This condition is also considered as present in *Parnaibaia* based on the coding of this character by Dutel et al.[24] (a coding re-used in most subsequent analyses). But the figure of *Parnaibaia* provided by Yabumoto (2008)[33] shows the snout with loosely connected small ossifications. Based on this short discussion, we provided in our analysis a new state definition ('consolidated, edentulous') as the derived state 1 of character 2 ('snout bones lying free versus consolidated'). We coded it as uncertain for *Laugia*, as plesiomorphic for *Parnaibaia* and as derived in *Megalocoelacanthus*. Moreover, we defined a second derived sate, 'consolidated, toothed', for *Macropoma* and *Swenzia*.

The condition of the supraorbital canal running in an open wide groove in *Foreyia* is unique among actinistians. In *Megalocoelacanthus* and *Libys*, two latimeriid coelacanths, the supraorbital sensory canal also runs in large grooves but there the groove is bridged by bony pillars, which define large oval openings[24]. The unique condition present in *Foreyia* necessitates the definition of a new character state, 'supraorbital sensory canals opening through bones as a large continuous groove without pillars' (char. 23 [4]).

Forey's character [51] reads 'lachrymojugal sutured to preorbital and lateral rostral (0) or lying in sutural contact with the tectal-supraorbital series (1)'. This character is intimately associated to character [10] 'preorbital absent (0) or present (1)' (character state 51 [0] associated with character state 10 [1] and vice versa). The only exception are genera which lack preorbital (10 [0]) and have a lachrymojugal sutured with the lateral rostral (51[0]), such as *Coelacanthus* and *Mawsonia*. In these taxa, the main difference with other genera with no preorbital is that there is a gap between the anterior extremity of the lachrymojugal and the supraorbital-tectal series. But in this case, *Latimeria*

and *Macropoma* should have been coded (0) as well, while they were coded in previous analyses (1). We suggest redefining character [51] in a more straightforward way as following: 'contact between the lachrymojugal and the preorbital or tectal-supraorbital series present (0) absent (1)'. Based on the literature, we re-coded the character for *Chinlea*[34], *Diplurus*[13,35], *Holophagus* and *Undina*[36] (*Holophagus* by comparison with *Undina*), *Axelrodichthys*[13], *Garnbergia*[37] and *Parnaibaia*[33].

In *Foreyia*, the teeth on the fourth coronoid show a gradation in size with the largest one in the mid-length of the bone. The lateral edge of the coronoid appears to roll over the dentary process. Also, the teeth borne on the coronoids are conical and cannot be considered as villiform. This condition is regarded as corresponding to the derived state of Forey's characters [56] and [67], even if the teeth remain small and not exactly fang-like.

Characters definitions

1. **Intracranial joint margin:**
   0. straight
   1. strongly interdigitate
2. **Snout bones:**
   0. lying free from one another
   1. consolidated, edentulous
   2. consolidated, toothed
3. **Median rostral:**
   0. single
   1. several median rostrals (internasals)
4. **Premaxillae:**
   0. paired
   1. fragmented
5. **Premaxilla:**
   0. with dorsal lamina
   1. without dorsal lamina
6. **Anterior opening of the rostral organ contained:**
   0. within premaxilla
   1. within separated rostral ossicles
7. **Parietal:**
   0. one pair
   1. two pairs
8. **Anterior and posterior pairs of parietals:**
   0. of similar size
   1. of dissimilar size
9. **Number of supraorbitals/tectals:**
   0. fewer than eight
   1. more than 10

10. **Preorbital:**
- 0. absent
- 1. present

11. **Parietal descending process:**
- 0. absent
- 1. present

12. **Intertemporal:**
- 0. absent
- 1. present

13. **Postparietal descending process:**
- 0. absent
- 1. present

14. **Supratemporal descending process:**
- 0. absent
- 1. present

15. **Extrascapulars:**
- 0. sutured with postparietals
- 1. free

16. **Extrascapulars:**
- 0. behind level of neurocranium
- 1. forming part of the skull roof

17. **Number of extrascapulars:**
- 0. three
- 1. five
- 2. more than seven

18. **Posterior margin of the skull roof:**
- 0. straight
- 1. embayed

19. **Supraorbital sensory canal:**
- 0. running through centre of ossification
- 1. following sutural course

20. **Medial branch of otic canal:**
- 0. absent
- 1. present

21. **Otic canal:**
- 0. joining supratemporal canal within lateral extrascapular
- 1. in supratemporal

22. **Anterior branches of supratemporal commissure:**
- 0. absent
- 1. present

23. **Supraorbital sensory canals opening through bones:**
- 0. as single large pores
- 1. bifurcating pores

2. many tiny pores
3. a large, continuous groove crossed by pillars
4. a large, continuous groove without pillars

24. Anterior pit line:
0. absent
1. present

25. Middle and posterior pit lines:
0. within posterior half of postparietals
1. within anterior third

26. Pit lines:
0. marking postparietals
1. not marking postparietals

27. Parietals and postparietals:
0. ornamented with enamel-capped ridges/tubercles
1. bones unornamented
2. bones marked by coarse rugosities

28. Parietals and postparietals:
0. without raised areas
1. with raised areas

29. Cheek bones:
0. sutured to one another
1. separated from one another

30. Spiracular (postspiracular):
0. absent
1. present

31. Preoperculum:
0. absent
1. present

32. Suboperculum:
0. absent
1. present

33. Quadratojugal:
0. absent
1. present

34. Squamosal:
0. limited to the mid-level of cheek
1. extending behind the postorbital to reach the skull roof

35. Lachrymojugal:
0. not expanded anteriorly
1. expanded anteriorly

36. Lachrymojugal:
0. ending without anterior angle
1. angled anteriorly

37. **Squamosal:**
0. large
1. reduced to a narrow tube surrounding the jugal sensory canal only

38. **Preoperculum:**
0. large
1. reduced to a narrow tube surrounding the preopercular canal only

39. **Preoperculum:**
0. undifferentiated
1. developed as a posterior tube-like canal-bearing portion and an anterior blade-like portion

40. **Postorbital:**
0. simple, without anterodorsal excavation
1. anterodorsal excavation in the postorbital

41. **Postorbital:**
0. without anterior process
1. with anterior process

42. **Postorbital:**
0. large
1. reduced to a narrow tube surrounding the sensory canal only

43. **Postorbital:**
0. entirely behind the level of the intracranial joint
1. spanning the intracranial joint

44. **Infraorbital canal within the postorbital:**
0. with simple pores opening directly from the main canal
1. anterior and posterior branches with the postorbital

45. **Infraorbital sensory canal:**
0. running through centre of postorbital
1. running at the anterior margin of the postorbital

46. **Jugal sensory canal:**
0. simple
1. with prominent branches

47. **Jugal canal:**
0. running through centre of bone
1. running along the ventral margin of the squamosal

48. **Pit lines:**
0. marking cheek bones
1. failing to mark cheek bones

49. **Ornaments upon cheek bones:**
0. absent
1. tubercular
2. represented as a coarse superficial rugosity

50. **Infraorbital, jugal and preopercular sensory canals:**
0. opening through many tiny pores

1. opening through a few large pores
2. a large, continuous groove crossed by pillars

51. Contact between the lachrymojugal and the preorbital or tectal-supraorbital series:
New definition for this character
0. present
1. absent

52. Sclerotic ossicles:
0. absent
1. present

53. Retroarticular and articular:
0. co-ossified
1. separated

54. Dentary teeth:
0. fused to the dentary
1. separated from dentary

55. Number of anterior coronoids:
0. 0
1. 1
2. 2
3. 3
4. 4

56. Coronoid:
0. opposite to the posterior end of dentary not modified
1. modified

57. Dentary:
0. simple
1. dentary hook-shaped

58. Oral pit line:
0. confined to angular
1. oral pit line reaching forward to the dentary and/or the splenial

59. Oral pit line:
0. located at centre of ossification of angular
1. removed from centre of ossification

60. Subopercular branch of the mandibular sensory canal:
0. absent
1. present

61. Dentary sensory pore:
0. absent
1. present

62. Ornaments:
0. ridged
1. granular

63. Dentary:

0. with ornament
1. without ornament

64. Splenial:
0. with ornament
1. without ornament

65. Dentary:
0. without prominent lateral swelling
1. with swelling

66. Principal coronoid:
0. lying free
1. sutured to angular

67. Coronoid fangs:
0. absent
1. present

68. Prearticular and/or coronoid teeth:
0. pointed and smooth
1. rounded and marked with fine striations radiating from the crown
2. pointed and marked with fine striations

69. Orbitosphenoid and basisphenoid regions:
0. co-ossified
1. separate

70. Optic foramen:
0. enclosed by basisphenoid extending forward
1. lying within separate interorbital ossification or cartilage

71. Processus connectens:
0. failing to meet parasphenoid
1. meeting parasphenoid

72. Basipterygoid process:
0. absent
1. present

73. Antotic process:
0. not covered by parietal descending process
1. covered

74. Temporal excavation:
0. lined with bone
1. not lined

75. Otico-occipital:
0. solid
1. separated to prootic/opisthotic

76. Supraoccipital:
0. absent
1. present

77. Vestibular fontanelle:

0. absent
1. present

78. Buccohypophysial canal:
0. closed
1. opening through parasphenoid

79. Parasphenoid:
0. without ascending laminae anteriorly
1. with ascending laminae

80. Suprapterygoid process:
0. absent
1. present

81. Vomers:
0. not meeting in the midline
1. meeting medially

82. Prootic:
0. without complex suture with the basioccipital
1. with a complex suture

83. Superficial ophthalmic branch of anterodorsal lateral line nerve:
0. not piercing antotic process
1. piercing antotic process

84. Process on braincase for articulation of infrabranchial 1:
0. absent
1. present

85. Separate lateral ethmoids:
0. absent
1. present

86. Separate basioccipital:
0. absent
1. present

87. Dorsum sellae:
0. small
1. large and constricting entrance to cranial cavity anterior to the intracranial joint

88. Extracleithrum:
0. absent
1. present

89. Anocleithrum:
0. simple
1. forked

90. Posterior neural and haemal spines:
0. abutting one another
1. not abutting

91. Occipital neural arches:
0. not expanded

1. expanded

92. Ossified ribs:

0. absent
1. present

93. Diphycercal tail:

0. absent
1. present

94. Fin rays:

0. more numerous than radials
1. equal in number

95. Fin ray:

0. branched
1. unbranched

96. Fin rays in D1:

0. > 10
1. 8-9
2. < 8

97. Caudal lobes:

0. symmetrical
1. asymmetrical

98. D1:

0. without denticles
1. with denticles

99. Paired fin rays:

0. not expanded
1. expanded

100. Pelvics:

0. abdominal
1. thoracic

101. Basal plate of D1:

0. with smooth ventral margin
1. emarginated and accommodating the tips of adjacent neural spines

102. D2 basal support:

0. simple
1. forked anteriorly

103. Median fin rays:

0. not expanded
1. expanded

104. Scale ornament:

0. not differentiated
1. differentiated

105. Lateral line openings in scales:

0. single
1. multiple

106. Scales:

0. ornament of ridges or tubercles
1. rugose

107. Ossified bladder:

0. absent
1. present

108. Pelvic bones of each side:

0. remain separate
1. fused in midline

109. Ventral keel scales:

0. absent
1. present

110. Ventral swelling of the palatoquadrate:

0. absent
1. present

Datamatrix

Actinopterygians	?0000?0??0010000?00000211?00001111???00????????010?1004000?0000000000??1??00110000010000?100000000000000000000
Porolepiforms	00100?0??0000000000000210?00011111??0000000000001001003000?00?0000100101?000?101000?0?0000000000?0000000000000
Diplocercides	001???110100000000100021100001110100000000000000010010030010000000000011101001101?010000??00010100000??0000???0
Rhabdoderma	100000100110010010110000100001110000000000001100100101410000100100101110110?0000?00010110100111000001100001000
Caridosuctor	1000001101?0??001011000010000111??000000000??0001001014100001010001????????????????????101?0111010001100?0100?
Hadronector	00100011?1?0??000010001010000111 00??0000000?1?0?10010???0010000000?????????????????????101?011100000010??01000
Rebellatrix	??1?????????1????????????????????????????1011011100000010000?0??

Polyosteorhynchus	00?0001101???100?010101???000111?0000000000?1?0010010 1?0001010??000????????????????????1?110111?1000100?0 1000
Allenypterus	0?????1001?0??000010000000001?110000000001001??101010 1??0110001000????????????????????10100101010000000000 1010
Lochmocercus	??????????????????10100???000111??00000000?01000?001? 0?00??000??000????????????????????101??10100000100??0 ?000
Coelacanthus	00?11?110010010011?110?0?1101???00001??0010?1?1?10010 1410??01?110000???????????????????10100111010000100?0 1000
Spermatodus	1000001101100110?111?020?1001?110100000000????010010 141?000?1???01110101??0110??00?101???????????????0?0 ????
Whiteia	00?000100110011011111000100011110001000000001110010011 141101?1111000001000111??00?11011111?10011120100010010 0000
Laugia	1??0??0?0110010010110000?100100000000000000001001110010 14000001111000011101110110??00010110110111110110100?0 1100
Sassenia	10????1?01100100?01??0201000111101000000000010001001? 1?10??0111100??011011001?01?0011011???????????????0?0 ??00
Chinlea	00?01?100010??0011?110????2011100?110000001?0???2000? ??11???1?11001????????????????????1?1?11111?000?101?1 ?00?
Diplurus	00001?111010101021111000?11011100010000000100111101001 ??000001?11000010001?11?00???0?11110111111101000101?0 0000
Holophagus	10?01?1110101110?1111?20?100101100100000000?1??110001 ??110011110001???????11?????1??1??1?1101111?1100110?0 100?
Undina	00?00?1110101110211?1?2???01101100??0000000?101110001 ??1101?1100001010001011?01??1??111101?011110100010010 1001
Coccoderma	10?011110?10010010110020101010100?001100000100110001? 14100001111000????????????????????1111011100011010011 1100
Libys	0???1?????10111??1111?30?110101000100010000?1???02?0? 14110011?110001???????????????????1111011110110011010 1001

Mawsonia	1?????110010100111?11020?12010?000100100101?0001200011??1??01?111001?????????????????????1??111?01000101?110?0
Macropoma	02?0?1111010111021111120?100101?00100011000110111010114?10111111000010001011?01?110?11111110111201000101101001
Latimeria	00111111101011102111110010211111001000110001111111101141101111?1100101000101100101100111101101112010001001000011
Miguashaia	00?0000?0?01??000000002?00000?1101??00000000000?10?1?0??001??0??0100???????????????????1?0??0000?000??0000??0?
Axelrodichthys	1000111100101001111?1020?1201010001011001011000120?011401??01?111?0110001111?0??110?1111?111111101000101?11000
Holopterygius	?0?0????????????????????0????????????????????????????00??0?????????00?????????00????????10?00101?1????00???1?
Garnbergia	??????11?0?0?????1??????????1?10001100?0000?????1?00???1?0??0100?0??0?
Parnaibaia	0011??1000?0??0011?1??2??110111000110000001?????1?001??01???11111?00???????????????????1?1?1111101000?01?010??
Swenzia	01?0??1????011???1??11?01011111?001000110001101110?0?1??101?1?110??0??????????1??????????1?011??0??001?0?01???
Guizhoucoelacanthus	0000??1001?0??000111100000101?1?0001000000101?001000????000?1111000????????????????????1?1001102000001000 1??0?
Piveteauia	1?????????1011?0?1?1?1?0??00?11??0000???????01011??111??0???1???00?0?????????11????????1??10??1110010100?01?0?
Euporosteus	0?1??010?10?0?10?0??0?0???????00?????????????????????????????????????011?0????1010?1?0?0????????????????1????1?
Axelia	??????0?0110?110211?100???00???????????????????????1????0???1????001??????????????????????????????0????0?0???0
Wimania	??????0???1001??????????????1?0???01000?00????0???01???10??????????0???????????????????????0???????????0?0???0

Megalocoelacanthus	01?0011100101110?1111?30??101????????????????????2?011?01??11?11000111001 11??01???0?1?11??1???????????????????1
Luopingcoelacanthus	???01?110??01?10?1???????1001?100001000000???00110?111??1???1111000?100???????1??????1?10100111001000101?0100?
Yunnancoelacanthus	??00??1001?0110101?????001001?10?010000000?????11?0??0??0??0?11??002????????????????1????1001?11?100?101?0?00?
Dobrogeria	1?????????101110?1111??0?1001??1??11????????????1???1????01?1??1????1010111???????0??111?????????????????????0
Indocoelacanthus	0?????1????0????????????????1???00??000000??????10?0????1?????1000?????????????????????????????????????0?0????
Lualabea	0??????????0???1??1?????111???
Ticinepomis	0??0??10??10?????0????????001?1?0???000?011????????0?131???11?110010?????????????????1?1?1?0111101000110000001
Foreyia	00??111001?0??????1???4???0010110000000101100??11000?1411???11110010??0???????1?????1??101?011100100??11?00??1

Results

We obtained 259 most parsimonious trees of 317 steps (Fig. S8); Consistency index (CI) = 0.3817; Homoplasy index (HI) = 0.6183; CI excluding uninformative characters = 0.3797; HI excluding uninformative characters = 0.6203; Retention index (RI) = 0.6766; Rescaled consistency index (RC) = 0.2582.

E – Heterochronic development in *Foreyia* and outline of its potential genetic roots

Development

Foreyia differs from the general coelacanth anatomy, and especially from its nearest relatives *Ticinepomis*, by its hypertrophied otico-occipital skull area and clavicle, its modified mandible, the dense covering of large tubercles, its very short portion of the vertebral column, its large unpaired fins and reduced paired fins. Tetrapods differ from 'fishes' by the presence of a neck, i.e. free cervical vertebrae that separate the skull from the pectoral girdle. In chondrichthyans and actinopterygians, the posttemporal, supracleithrum and/or anocleithrum bones make a connection between the skull and the pectoral girdle. Coelacanths are, in a way, intermediate between 'fishes' and tetrapods by having a gap between the skull and the pectoral girdle. The morphological peculiarities of the occipital and pectoral regions of *Foreyia*, i.e. the hypertrophied occipital region and clavicle bone, and the close spatial relationship between the pectoral girdle and skull, provide an intriguing evo-devo issue in connection with the neck development. Most of these unusual features have their developmental origin at the level of the skull – trunk interface region in the embryo. One of the more conserved gnathostoms muscles of the neck region is the *cucullaris* muscle. The *cucullaris* muscle was considered absent in coelacanths[13,38], but Sefton et al.[39] recently identified it in *Latimeria* by re-interpreting the fifth gill *levator*, a muscle which originates on the anocleithrum and extends on the fifth ceratobranchial. The *cucullaris* muscle originates from the occipital lateral plate mesoderm at the level of somites 1-3 and shows a genetic pathway more similar to the head muscles rather than to the trunk muscles pathway[40]. The same lateral plate mesoderm forms the ventromedial extremity of the clavicle in amniotes[41,42]. The dermal clavicle of bony fishes, including coelacanths, is regarded as homologous to the partly endochondral clavicle of mammals based on the scaffold model[41]. This model proposes that muscles connectivity in the neck region is homologous structures in all gnathostoms, and that the dermal versus enchondral modes of ossification are not indicative of the embryological origin. In the chick, the first somites region also gives rise to part of the otic capsule and to the exoccipital (somite 1), and to the basioccipital (somites 2-4) bones[43]. More anteriorly, the paraxial mesoderm of the hindbrain gives rise to part of the otic capsule and to the supraoccipital, while the paraxial mesoderm of the mid-brain gives rise to the sphenoid[43]. The occipital, otic and sphenoid ossifications form the 'chordal skull' according to Couly et al.[43]. If we restrict the 'chordal skull' to the occipital and otic region, i.e. those originating from the somitic and hindbrain paraxial mesoderm, it corresponds to the independent otico-occipital portion of the braincase in coelacanths. The 'prechordal skull' of Couly et al.[43] has its origin in the neural crest. When we add the sphenotic ossifications, originating in the midbrain paraxial mesoderm, to the 'prechordal skull' of Couly et al.[43], it corresponds to the independent ethmo-sphenoid portion of coelacanths. Consequently, most of the highly derived skeletal components in *Foreyia* have their embryological origin at the anterior extremity of the trunk region.

Genetic pathways

We hypothesize here that the derived characters of *Foreyia* compared to other coelacanths, and especially to its sister-genus *Ticinepomis* are the result of evolution caused by a heterochronic

developmental program. Because most of the modified features have their origin in a concentrated region of the embryo as seen above, little changes in the expression of a developmental patterning gene may cause dramatic alterations in the adult phenotype. The patterning role of the *Hox* genes, in particular of *HoxD4*, which control the mesoderm at the level of the skull-trunk boundary in mouse[41,44] is a candidate since we know that the *Hox* clusters in *Latimeria* are very similar compared to other species with four clusters including mammals[45]. *Hox* genes are also preponderant in the building and regionalization of the vertebral axial skeleton[46], which is autapomorphic in *Foreyia*. *Prrx1* (former *MHox* or *Prx1*) and *Prrx2* (former *S8* or *Prx2*) are transcriptional regulators during the developmental processes. Phenotypic modifications caused by changes of expressions of *Prrx1* / *Prrx2* affect mostly the cranial neural crest corresponding to the first branchial arch and, to a lesser degree, to structures that arise from the second branchial arch and to the cephalic mesoderm[47,48]. Some of the affected anatomical parts correspond to some of the atypical features of *Foreyia*, such as modified mandible and palate, which originated from the cranial crest of the first arch, and modified supraoccipital, which originated from the lateral cephalic mesoderm. The tissues at the neck/trunk transition, which give rise to the most modified components in *Foreyia*, however, seem lightly under the control of these genes, at least in mice. *Tbx15*, which encodes transcription factors, is expressed in the limb buds, branchial arches, flanks and the craniofacial region[49]. Some of the phenotypic malformations observed in the *Tbx15* mutant mice concern equivalent modified components parts in *Foreyia*, such as changes in size of some dermal and enchondral skull bones and limb bones, as well as malformations on the anterior vertebrae and on the scapula (in particular the acromion). This gene, which has a complementary expression pattern with *Pax1* discussed below[50], might be involved in the modified anatomical components of *Foreyia*. *Pax* genes, which are transcriptions factors that play important role in embryonic development, are other putative candidates. The nine *Pax* genes, which represent the basic set in vertebrates, are present in *Latimeria*[51]. *Pax9* codes for transcription factors required for, among others, the development of teeth, skeletal elements of skull and larynx as well as distal limbs[51,52]. In the chondrichthyans and the amniotes, the embryonic expression of *Pax9* occurs at the level of the head mesoderm, the sclerotomes of the somites (first ones give rise to the occipital bones), at the level of the postotic (gives rise to the clavicle) and lateral mesoderms (gives rise to paired limbs), as well as at the level of the neural crest (gives rise to odontodes)[53,54]. *Pax1* has a more localised expression in the pharyngeal pouches, the sclerotome, the postotic lateral and lateral mesoderm, but not in the head mesoderm[54-56]. Mice that are mutant for *Pax1* gene have an abnormal acromion. This process of the scapula is mesodermal in origin[41] as is the hypertrophied clavicle of *Foreyia*. Considering that gene expression of *Pax1* and *Pax9* may have been more similar to each other in coelacanths than in amniotes, as it is the case in the ray-fin fish Medaka[57], both might have acted together.

1 Bürgin, T., Eichenberger, U., Furrer, H. & Tschanz, K. Die Prosanto-Formation—eine fischreiche Fossil-Lagerstätte in der Mitteltrias der Silvretta-Decke (Kanton Graubünden, Schweiz. *Eclogae geol. Helv.* **84**, 921-990 (1991).

2 Furrer, H., Eichenberger, U., Froitzheim, U. & Wurster, D. Geologie, Stratigraphie und Fossilien der Ducankette und des Landwassergebiets (Silvretta-Decke, Ostalpin). *Eclogae geol. Helv.* **85**, 245–256 (1992).

3 Furrer, H. The Prosanto Formation, a marine Middle Triassic Fossil-Lagerstätte near Davos (Canton Graubünden, Eastern Swiss Alps). *Eclogae geol. Helv.* **88**, 681-683 (1995).

4 Grauvogel-Stamm, L., Meyer-Berthaud, B. & Vozenin-Serra, C. Conifer axes from the Middle Triassic of Switzerland: structure and affinities. *Courier-Forschunginstitut Senckenberg*, 51-68 (2003).

5 Furrer, H. *So kam der Fisch auf den Berg: eine Broschüre über die Fossilfunde am Ducan*. (Bündner Naturmuseum, 2009).

6 Scheyer, T. M. & Desojo, J. B. Palaeohistology and external microanatomy of rauisuchian osteoderms (Archosauria: Pseudosuchia). *Palaeontology* **54**, 1289-1302 (2011).

7 Fraser, N. & Furrer, H. A new species of *Macrocnemus* from the Middle Triassic of the eastern Swiss Alps. *Swiss J. Geosci.* **106**, 199-206 (2013).

8 Furrer, H., Schaltegger, U., Ovtcharova, M. & Meister, P. U-Pb zircon age of volcaniclastic layers in Middle Triassic platform carbonates of the Austroalpine Silvretta nappe (Switzerland). *Swiss J. Geosci.* **101**, 595-603 (2008).

9 Mundil, R., Brack, P., Meier, M., Rieber, H. & Oberli, F. High resolution U-Pb dating of Middle Triassic volcaniclastics: Time-scale calibration and verification of tuning parameters for carbonate sedimentation. *Earth Planet. Sc. Lett.* **141**, 137-151 (1996).

10 Stockar, R., Baumgartner, P. O. & Condon, D. Integrated Ladinian bio-chronostratigraphy and geochrononology of Monte San Giorgio (Southern Alps, Switzerland). *Swiss J. Geosci.* **105**, 85-108 (2012).

11 Cavin, L., Furrer, H. & Obrist, C. New coelacanth material from the Middle Triassic of eastern Switzerland, and comments on the taxic diversity of actinistans. *Swiss J. Geosci.* **106**, 161-177 (2013).

12 Ørvig, T. in *Problems in Vertebrate Evolution* (eds S. M. Andrews, R. S. Miles, & A. D Walker) 53-75 (Academis Press, 1977).

13 Forey, P. L. *History of the Coelacanth Fishes*. 419 (Chapman and Hall, London, 1998).

14 Long, J. A. Cranial ribs in Devonian lungfishes and the origin of air-breathing. Memoir of the Australasian *Association of Palaeontologists* **15**, 199-210 (1993).

15 Clement, A. M. & Long, J. A. Air-breathing adaptation in a marine Devonian lungfish. *Biol. Lett.*, doi:10.1098/rsbl.2009.1033 (2010).

16 Clement, A. M. A new species of long-snouted lungfish from the Late Devonian of Australia, and its functional and biogeographical implications. *Palaeontology* **55**, 51-71 (2012).

17 Clement, A. M., Long, J., Tafforeau, P. & Ahlberg, P. E. The dipnoan buccal pump reconstructed in 3D and implications for air breathing in Devonian lungfishes. *Paleobiology* **42**, 289-304 (2016).

18 Cupello, C., Clément, G. & Brito, P., M. in *Evolution and Development of Fishes* (eds Z. Johanson, C. Underwood, & M Richter) (Cambridge University Press, in press).

19 Pardo, J. D., Huttenlocker, A. K. & Small, B. J. An exceptionally preserved transitional lungfish from the Lower Permian of Nebraska, USA, and the origin of modern lungfishes. *PLoS ONE* **9**, e108542, doi:10.1371/journal.pone.0108542 (2014).

20 Kemp, A., Cavin, L. & Guinot, G. Evolutionary history of lungfishes with a new phylogeny of post-Devonian genera. *Palaeogeogr. Palaeocl.* **471**, 209-219 (2017).

21 Brito, P. M., Meunier, F., Clément, G. & Geffard-Kuriyama, D. The histological structure of the calcified lung of the fossil coelacanth *Axelrodichthys araripensis* (Actinistia: Mawsoniidae). *Palaeontology* **53**, 1281-1290, doi:10.1111/j.1475-4983.2010.01015.x (2010).
22 Cupello, C. et al. Allometric growth in the extant coelacanth lung during ontogenetic development. *Nature communications* **6** (2015).
23 Cupello, C., Meunier, F. J., Herbin, M., Clément, G. & Brito, P. M. Lung anatomy and histology of the extant coelacanth shed light on the loss of air-breathing during deep-water adaptation in actinistians. *R.Soc.open sci.* **4**, 161030 (2017).
24 Dutel, H. *et al.* The giant Cretaceous coelacanth (Actinistia, Sarcopterygii) *Megalocoelacanthus dobiei* Schwimmer, Stewart & Williams, 1994, and its bearing on Latimerioidei interrelationships. *PLoS ONE* **7**, e49911 (2012).
25 Rieppel, O. A new coelacanth from the Middle Triassic of Monte San Giorgio, Switzerland. *Eclogae geol. Helv.* **73**, 921-939 (1980).
26 Cloutier, R. *The primitive actinistian Miguashaia bureaui Schultze (Sarcopterygii).* (Verlag Dr. Friedrich Pfeil, München, 1996).
27 Forey, P. L., Ahleberg, P. E., Luksevics, E. & Zupins, I. A new coelacanth from the Middle Devonian of Latvia. *J. Vertebr. Paleontol.* **20**, 243-252 (2000).
28 Jarvik, E. *Middle and Upper Devonian Porolepiformes from East Greenland with Special Reference to* Glyptolepis groenlandica *N. Sp: And a Discussion on the Structure of the Head in the Poroplepiformes*. (CA Reitzel, 1972).
29 Cloutier, R. Patterns, trends, and rates of evolution within the Actinistia. *Environ. Biol. Fish.* **32**, 23–58 (1991).
30 Cavin, L. & Gradinăru, E. *Dobrogeria aegyssensis*, a new early Spathian (Early Triassic) coelacanth from North Dobrogea (Romania). *Acta Geol. Pol.* **64** 139-165 (2014).
31 Clément, G. A new coelacanth (Actinistia, Sarcopterygii) from the Jurassic of France, and the question of the closest relative fossil to *Latimeria*. *J. Vertebr. Paleontol.* **25**, 481-491 (2005).
32 Stensiö, E. A. Triassic fishes from East Greenland, collected by the Danish expeditions in 1929-1931. *Meddelelser om Grønland* **Band 83**, 1-305 (1932).
33 Yabumoto, Y. A New Mesozoic coelacanth from Brazil (Sarcopterygii, Actinistia). *Paleontol. Res.* **12**, 329-343 (2008).
34 Schaeffer, B. *Late Triassic fishes from the western United States*. (American Museum of Natural History, 1967).
35 Schaeffer, B. The Triassic coelacanth fish *Diplurus*, with observations on the evolution of the Coelacanthini. *B. Am. Mus. Nat. Hist.* **99**, 25-78 (1952).
36 Saint-Seine, P. d. Les poissons des calcaires lithographiques de Cerin (Ain). *Nouvelles archives du musée d'histoire naturelle de Lyon* **2**, 1-357 (1949).
37 Martin, M. & Wenz, S. Découverte d'un nouveau Coelacanthidé, *Garnbergia ommata* n.g., n.sp., dans le Muschelkalk supérieur du Baden-Würtenberg. *Stuttgarter Beitr. Naturk., Ser. B* **105**, 1-17 (1984).
38 Ericsson, R., Knight, R. & Johanson, Z. Evolution and development of the vertebrate neck. *J. Anat.* **222**, 67-78 (2013).
39 Sefton, E. M., Bhullar, B.-A. S., Mohaddes, Z. & Hanken, J. Evolution of the head-trunk interface in tetrapod vertebrates. *eLife* **5**, e09972 (2016).

40 Theis, S. *et al.* The occipital lateral plate mesoderm is a novel source for vertebrate neck musculature. *Development* **137**, 2961-2971 (2010).

41 Matsuoka, T. *et al.* Neural crest origins of the neck and shoulder. *Nature* **436**, 347-355 (2005).

42 Nagashima, H. *et al.* Developmental origin of the clavicle, and its implications for the evolution of the neck and the paired appendages in vertebrates. *J. Anat.* **229**, 536-548 (2016).

43 Couly, G. F., Coltey, P. M. & Le Douarin, N. M. The triple origin of skull in higher vertebrates: a study in quail-chick chimeras. *Development* **117**, 409-429 (1993).

44 Santagati, F. & Rijli, F. M. Cranial neural crest and the building of the vertebrate head. *Nat. Rev. Neurosci.* **4**, 806-818 (2003).

45 Amemiya, C. T. *et al.* The African coelacanth genome provides insights into tetrapod evolution. *Nature* **496**, 311-316 (2013).

46 Casaca, A., Santos, A. C. & Mallo, M. Controlling Hox gene expression and activity to build the vertebrate axial skeleton. *Dev. Dynam.* **243**, 24-36 (2014).

47 Martin, J. F., Bradley, A. & Olson, E. N. The *paired*-like homeo box gene *MHox* is required for early events of skeletogenesis in multiple lineages. *Gene. Dev.* **9**, 1237-1249 (1995).

48 ten Berge, D., Brouwer, A., Korving, J., Martin, J. F. & Meijlink, F. *Prx1* and *Prx2* in skeletogenesis: roles in the craniofacial region, inner ear and limbs. *Development* **125**, 3831-3842 (1998).

49 Singh, M. K. *et al.* The T-box transcription factor *Tbx15* is required for skeletal development. *Mech. develop.* **122**, 131-144 (2005).

50 Kuijper, S. *et al.* Genetics of shoulder girdle formation: roles of *Tbx15* and aristaless-like genes. *Development* **132**, 1601-1610 (2005).

51 Paixão-Côrtes, V. R., Salzano, F. M. & Bortolini, M. C. Evolutionary history of chordate *PAX* genes: dynamics of change in a complex gene family. *PloS ONE* **8**, e73560 (2013).

52 LeClair, E. E., Bonfiglio, L. & Tuan, R. S. Expression of the paired-box genes *Pax-1* and *Pax-9* in limb skeleton development. *Dev. Dynam.* **214**, 101-115 (1999).

53 Peters, H., Neubüser, A., Kratochwil, K. & Balling, R. *Pax9*-deficient mice lack pharyngeal pouch derivatives and teeth and exhibit craniofacial and limb abnormalities. *Gene. Dev.* **12**, 2735-2747 (1998).

54 Adachi, N., Takechi, M., Hirai, T. & Kuratani, S. Development of the head and trunk mesoderm in the dogfish, *Scyliorhinus torazame*: II. Comparison of gene expression between the head mesoderm and somites with reference to the origin of the vertebrate head. *Evol. Dev.* **14**, 257-276 (2012).

55 Timmons, P. M., Wallin, J., Rigby, P. & Balling, R. Expression and function of *Pax 1* during development of the pectoral girdle. *Development* **120**, 2773-2785 (1994).

56 Wilm, B., Dahl, E., Peters, H., Balling, R. & Imai, K. Targeted disruption of *Pax1* defines its null phenotype and proves haploinsufficiency. *P. Natl. Acad. Sci. USA* **95**, 8692-8697 (1998).

57 Mise, T., Iijima, M., Inohaya, K., Kudo, A. & Wada, H. Function of *Pax1* and *Pax9* in the sclerotome of medaka fish. *Genesis* **46**, 185-192 (2008).

F – Supplementary Movie

Movie S1

Foreyia maxkuhni, gen. et sp. nov., surface CT image of the paratype (PIMUZ A/I 4372).
